# The Positive Influence of Individual-Level Disaster Preparedness on the Odds of Individual-Level Pandemic Preparedness—Insights from FEMA’s 2021–2023 National Household Survey

**DOI:** 10.3390/ijerph22050702

**Published:** 2025-04-29

**Authors:** Dionne Mitcham, Crystal R. Watson

**Affiliations:** 1 Johns Hopkins Center for Health Security, Johns Hopkins Bloomberg School of Public Health, Baltimore, MD 21202, USA; 2 Department of Environmental Health and Engineering, Johns Hopkins Bloomberg School of Public Health, Baltimore, MD 21205, USA

**Keywords:** pandemic preparedness, personal preparedness, pandemic, disaster, COVID-19

## Abstract

Objective: To explore the possible association and trends between individual-level disaster preparedness status (for natural, technological, and human-caused hazards) and the level of pandemic preparedness during the COVID-19 pandemic among adults in the U.S. from 2021 through 2023. Methods: Multivariate logistic regression was conducted using data from the U.S. Federal Emergency Management Agency’s (FEMA) annual National Household Survey (NHS) from 2021 to 2023 to identify statistically significant variables associated with personal pandemic preparedness behavior among participants of the nationally representative survey during the COVID-19 pandemic. Results: Overall, the results showed that the proportion of respondents that were considered prepared for a pandemic peaked in 2021 (54.0%) and steadily decreased in the following years (2022: 43.3%; 2023: 41.3%) highlighting the need for pandemic preparedness resources and educational campaigns to be available prior to a pandemic occurring. The final multivariate logistic regression models featuring consistent significant covariates demonstrated a highly statistically significant relationship between individual-level disaster preparedness and pandemic preparedness across all three study years (2021: odds ratio (OR): 21.35, standard error (SE): 2.59, *p* < 0.001; 2022: OR: 9.26, SE: 0.87, *p* < 0.001; 2023: OR: 6.75, SE: 0.59, *p* < 0.001). Conclusions: The significant results suggest individuals who are prepared for a disaster have higher odds of being prepared for a pandemic. These findings support the continued increase in collaboration between emergency management and public health entities to jointly support the development of evidence-based resources to increase personal preparedness for both disasters and pandemics.

## 1. Introduction

During the early stages of the COVID-19 pandemic, many individuals had to rely on their own personal preparedness to be resilient to pandemic-related health threats along with other concurrent challenges, such as severe weather events, amid a time of delayed assistance due to pandemic restrictions. Historically, the U.S. has had strong emergency preparedness programs, online resources, and campaigns to promote individual disaster preparedness for natural, technological, and human-caused events [1,2,3,4] (according to FEMA’s Guide for All-Hazard Emergency Operations, the term “disaster” is defined as “an occurrence of a natural catastrophe, technological accident, or human-caused event that has resulted in severe property damage, deaths, and/or multiple injuries” [4]). The disaster preparedness programs that provide these resources are historically supported at higher levels of government and carried out locally, but with little emphasis on pandemic preparedness. Despite the success of the individual disaster preparedness movement in the U.S., the number of programs and resources dedicated to individual disaster preparedness has yet to transfer to the realm of individual pandemic preparedness.

With the increased occurrence of epidemics and pandemics in the 21st century, there is a need to increase individual preparedness to bolster community resilience to the impacts of pandemics, both with and without other concurrent and cascading events (e.g., hurricanes, infrastructure failures, etc.) [5,6,7,8] (the revised International Health Regulations (as of 1 June 2024) define a “pandemic emergency” as “a public health emergency of international concern that is caused by a communicable disease and: (i) has, or is at high risk of having, wide geographical spread to and within multiple States; and (ii) is exceeding, or is at high risk of exceeding, the capacity of health systems to respond in those States; and (iii) is causing, or is at high risk of causing, substantial social and/or economic disruption, including disruption to international traffic and trade; and (iv) requires rapid, equitable and enhanced coordinated international action, with whole of-government and whole-of-society approaches ” [9]). Pandemic threats and natural disasters share similarities in preparedness mechanisms and their potential for societal disruption [10]. However, these two hazards can also differ widely in their context in which they occur, magnitude of impact, and duration of the event. In the literature, preparedness for these two types of events have often been addressed separately, both at the population level and at the personal-preparedness level. Although previous studies have demonstrated mixed-findings on the impact of individual preparedness on population-level disaster outcomes, personal preparedness has been shown to be essential for increasing self-sufficiency and individual resilience following a disaster [1,11,12]. The predictors and influencers of individual-level disaster preparedness behaviors have been well studied [13,14,15,16,17]. However, the current literature lacks longitudinal information on the predictors of individual-level pandemic preparedness, which could be attributed, at least in part, to the previous lack of data.

In 2021, the Federal Emergency Management Agency (FEMA) added pandemic-related questions to their annual nationally representative National Household Survey (NHS) to collect and assess pandemic preparedness behavior among U.S. adults [18]. Zhirui Chen and Zhen Cong novelly utilized the NHS pandemic-related data to investigate the influence of age and preparedness information on actual and perceived pandemic preparedness in 2021 [19]. Since 2021, FEMA has continued to collect pandemic preparedness data among U.S. participants. This study expands on Chen and Cong’s original investigation into predictors of individual-level pandemic preparedness behavior by applying similar statistical techniques to the NHS data from 2021 to 2023 to explore the possible relationship between disaster preparedness and pandemic preparedness, while determining whether pandemic preparedness status among U.S. adults altered in the years following the emergence of the COVID-19 pandemic. Overall, the study’s results could impact future programs and collaborations among the emergency management and public health fields to increase individual-level pandemic preparedness and indirectly bolster community resilience in the U.S.

## 2. Background

### 2.1. Individual-Level Preparedness in the U.S.

Although disaster preparedness is part of the remit of many agencies, FEMA is the U.S.’ main federal agency for disaster preparedness. FEMA provides state and local jurisdictions with programs, resources, and assistance to prepare for, respond to, and recover from disasters [20]. According to the Department of Homeland Security (DHS) and FEMA, preparedness is defined as the iterative process of “planning, organizing, training, equipping, exercising, evaluating, and taking corrective action” in order to have a conducive response and recovery to an incident [21]. Individual or personal preparedness is the concept of conducting preparedness activities, such as stockpiling and plan-making, on an individual-level to be prepared for a disaster. Often individual preparedness is utilized as a disaster management tool to promote self-sufficiency, safety, and resiliency within the initial 72 h of an incident for both individuals and communities at large [1].

Since 2003, FEMA has utilized the Ready.gov website and an accompanying educational campaign to promote individual disaster preparedness and provide educational preparedness resources for both adults and families [3]. Ready.gov provides the public with information accessible in multiple languages about how to make a preparedness kit, create a response plan, and stay informed during the stages of disaster [1,3,22]. In addition to Ready.gov, FEMA supports individual and community-based preparedness through a variety of programs such as the Community Emergency Response Team, faith-based community preparedness, and the Youth Preparedness Council [2]. Even though FEMA provides a variety of resources to support individual and community-level disaster preparedness, the agency’s efforts prior to 2020 were focused on natural weather-based disasters and technical disasters with a lack of focus on pandemic preparedness. According to FEMA’s historical disaster summary data, the incident type of “pandemic” had not been used before 2020 for a federal disaster declaration authorized under the Robert T. Stafford Disaster Relief and Emergency Assistance Act (the Stafford Act) [23].

Prior to the COVID-19 pandemic, many of the U.S. Department of Health and Human Services’ (HHS) agencies led and now continue to lead pandemic preparedness activities. Namely, the Centers for Disease Control and Prevention (CDC) has been and continues to be the lead HHS agency for promoting community and individual-level pandemic preparedness. Since 2002, the CDC has utilized their Public Health Emergency Preparedness (PHEP) cooperative agreement program to provide assistance to increase public health emergency preparedness activities at participating public health departments, including preparedness for pandemics [24]. In addition to the CDC’s efforts to increase public health preparedness at the local level, CDC utilizes health promotion campaigns and their website during a public health threat to promote event-specific information about individual-level response to epidemics and pandemics as seen during the 2009 H1N1 epidemic and the 2020 COVID-19 pandemic [25,26]. However, there remains a need for widely accessible individual-level pandemic preparedness resources and educational programs that are made available in advance of a pandemic.

### 2.2. Previous Work

In the field of disaster management, many studies have investigated the predictors of disaster preparedness behavior [13,14,15,16,17,27]. The commonality of this research could be attributed to both the availability of data and historical acceptance of disaster preparedness by society. Due to FEMA’s annual NHS, the agency can provide data on the tendencies and predictors of preparedness among U.S. Adults. Within the NHS, FEMA utilizes their four identified factors or “influencers” that influence an individual’s preparedness behaviors: awareness of disaster-related information, previous disaster experience, disaster-related self-efficacy, and disaster risk perception [28,29,30,31]. Prior to the 2021 NHS, FEMA only analyzed the influence of these items on an individual’s decision to prepare for a natural disaster or emergency. However, the addition of the pandemic-preparedness related questions to the general NHS survey in 2021 has enabled the ability to investigate the application of these four predictors of disaster preparedness as predictors for pandemic preparedness.

Since the start of the COVID-19 pandemic, there has been an increase in studies investigating the predictors of household or individual preparedness for a pandemic. In 2023, Clay et al. surveyed a cohort of 1020 New York City residents to identify the association of household preparedness in the areas of provision, practices, and planning with adaptions to the COVID-19 pandemic [32]. The study found mixed associations between the type of disaster preparedness conducted by individuals and their household characteristics. A similar study was conducted in 2020 by Ferreira et al. among a sample of 744 individuals living in the Southeastern Gulf Coast of the U.S. who experienced both a climate disaster and the COVID-19 pandemic [10]. By utilizing binary logistic regression, the authors identified higher levels of resilience (based on the scale), speaking English as a first language, and higher educational attainment were statistically significant explanatory variables (*p* < 0.1) for both disaster and pandemic preparedness behaviors. The variables of being in a relationship and identifying as white were also identified as strong predictors of disaster preparedness. Most notably, the study results revealed that individuals prepared for a disaster were more likely to also be prepared for a pandemic [10]. Despite the differing characteristics and consequences of natural disasters and pandemic threats, both hazards pose a risk of disrupting societal functioning. Given the ability for personal preparedness activities to aid in resiliency, the association between individual-level disaster and pandemic preparedness highlighted by Ferreira et al. warrants further investigation to determine whether this relationship is present within a nationally representative dataset.

As mentioned above, the 2024 paper by Chen et al. was the first published paper utilizing the NHS data to analyze predictors and trends of pandemic preparedness. However, the study by Chen et al. did not investigate the trajectory of the predictors of pandemic preparedness over multiple years as well as the relationship with disaster preparedness. Despite the enriching progress in individual-level pandemic preparedness literature, there is a persistent need to investigate on a national level the association between sociodemographic data, FEMA’s influencers of preparedness behaviors, and disaster preparedness status on the likeliness of U.S. adults to be prepared for a pandemic.

## 3. Methods

### 3.1. Data Source

This retrospective cross-sectional analysis utilized data from FEMA’s nationally representative cross-sectional NHS from 2021 to 2023. The NHS is conducted annually among randomly selected adults from all U.S. states and territories to assess the personal “preparedness actions, attitudes, and motivations” within the U.S. [33]. FEMA’s NHS survey requires each respondent to complete the core survey questions collecting information about “disaster preparedness, pandemic preparedness, demographics and other topics” through an online or phone survey conducted in English and Spanish [34]. In addition to utilizing a nationally representative sample, FEMA conducts oversampling for six hazard-specific outcomes that vary from year to year. Respondents from both the nationally representative and hazard-specific sampling schemes were included in this analysis, which is consistent with the sample selection made by Chen et al. [19]. For this analysis, FEMA’s provided geographic-based weighting scheme was utilized across the descriptive statistics and logistic regression analysis to maintain consistency among the analysis and ensure that the results remained nationally representative [34].

### 3.2. Study Population

In this study, we analyzed the available data from the NHS core survey questions for survey years (2021–2023) in which all participants were asked pandemic-preparedness-related questions in addition to the traditional disaster-related questions [18,35,36]. The unweighted datasets consisted of 7197 individuals in the 2021 survey year, 7145 in 2022, and 7604 in 2023. Participants were excluded from the study sample if they answered one or more questions corresponding with the covariates of interest with the replies of “unknown” or “don’t know”. Of the individuals in the unweighted NHS datasets, 9.8% (706), 13.4% (955), and 14.7% (1120) of participants were dropped from the respective datasets resulting in an unweighted sample of 6491 individuals for 2021, 6190 individuals for 2022, and 6484 individuals for 2023. After applying FEMA’s weighting core survey scheme, the overall weighted study population utilized for this analysis included 6321 individuals from the 2021 NHS dataset, 6171 from 2022, and 6404 participants from 2023.

### 3.3. Measures

The 2021–2023 NHS surveys were conducted by FEMA from 24 February 2021 to 14 May 2021; 10 February 2022 through 20 April 2022; and 1 February 2023 through 14 March 2023 [28,29,30]. Although the survey instruments had slight variation from year to year, the survey questions and list of responses corresponding to the covariates of interest remained consistent across all three study years, except for the covariates of previous pandemic experience, age, and total household annual income [18,35,36,37,38,39]. Below, we have described how the primary outcome variables, primary covariate, and possible confounding covariates were measured.

#### 3.3.1. Primary Outcome Variable

Within the NHS from 2021 to 2023, participants’ individual levels of pandemic preparedness were assessed via the question “What have you done to prepare for a pandemic in the last year?” [18]. Respondents were directed to select all applicable preparedness actions from the following options: “assemble or update supplies”, “document and insure property”, “get involved in your community”, “know evacuation routes”, “make a plan”, “make your home safer”, “plan with neighbors”, “practice emergency drills or habits”, “safeguard documents”, “save for a rainy day”, “sign up for alerts and warnings”, and “test family communication plan”. Respondents with two or less pandemic preparedness actions within the last year were categorized as unprepared for a pandemic, while respondents with at least three pandemic preparedness actions within the last year were categorized as prepared (0 = “unprepared”, 1 = “prepared”). This threshold for pandemic preparedness was selected due to the distribution of the count of preparedness actions per respondent and the consistent use of this cutoff within FEMA’s reporting summaries [28,29,30].

#### 3.3.2. Primary Covariate

Disaster preparedness was determined by the participants’ response to the question “What have you done to prepare for a disaster or emergency in the last year?” [18]. As with the primary outcome variable, the participants were provided with the same list of preparedness activities and their responses were dichotomized by the completion of three or more preparedness activities with the last year (0 = “unprepared”, 1 = “prepared”).

#### 3.3.3. Influencers of Preparedness

In the NHS survey from 2021 to 2023, the four influencers of preparedness were assessed in both the disaster and pandemic-related portions of the NHS questionnaire. This study analysis uniquely investigates the effect of these influencers on pandemic preparedness. However, information regarding the awareness of disaster preparedness information was included to compare yearly disaster and pandemic awareness trends from 2021 to 2023.

Within the pandemic preparedness section of the NHS, independent questions were used for each of the four preparedness influencers to investigate whether these disaster-based influencers were valid predictors of individual pandemic preparedness. Awareness of pandemic-related information was obtained by respondents selecting at least one of the information sources in response to the question “In the past year, what information did you read, see, or hear about how to better prepare for a pandemic?” [18]. Respondents selected from the answers of “avoiding infection”, “basic survival”, “planning/preparing”, “protecting yourself/family”, “risk categories”, “testing”, “treatment”, and “vaccines”. Awareness of pandemic-related information was utilized in our analysis as a dichotomized variable (0 = “no”, 1 = “yes”). Awareness of disaster-related information was assessed dichotomously by a similar question of “In the past year, what information have you read, seen, or heard about how to get better prepared for a disaster?” [18]. To assess the awareness of disaster preparedness information, participants were provided the same options utilized for the assessment of disaster preparedness actions completed in the last year.

In the 2021 NHS, previous pandemic experience was captured by combing the responses from the following dichotomous questions: (1) “Prior to COVID-19, had you or your family ever experienced the impacts of a pandemic?”; (2) “Have you or your family experienced the impacts of COVID-19?” [18]. However, in the 2022 and 2023 NHSs, previous pandemic experience was captured by the singular question of “Have you or your family ever experienced the impacts of a pandemic?” [35,39]. For this question, 2022 and 2023 survey respondents selected from the answers of “no”, “yes”, “yes, COVID-19”, and “yes, COVID-19 and something else” [35,36]. In our analysis, we combined pandemic experience responses into a dichotomized variable because of the scarce responses of “yes, COVID-19 and something else”. Pandemic-related self-efficacy was assessed through the question of “How confident are you that you can take steps to prepare for a pandemic in your area?” [18]. The following responses were handled as a categorical ordinal variable: “not at all confident”, “slightly confident”, “moderately confident”, “somewhat confident”, and “extremely confident”. Pandemic risk perception was gauged by the question of “Thinking about the area you live in, how likely would it be for a pandemic to impact you?” [18]. The responses were categorized into the ordinal variables of “unlikely”, “likely”, and “very likely”.

#### 3.3.4. Demographics

The FEMA NHS 2021–2023 asked respondents several demographic questions. However, we focused our interest on the demographics of race, age category, homeownership, education status, gender, total household annual income, census region, and rurality. Race was categorized into White, Black or African American, Asian, American Indian/Alaska Native, Native Hawaiian/Pacific Islander, other, and two or more races. In 2021, age was categorized into the age brackets of 18–29, 30–39, 40–49, 50–59, 60–69, 70–79, and over 80. However, in the 2022 and 2023 NHS, the age categories were alternatively defined as 18–19, 20–29, 30–39, 40–49, 50–59, 60–69, 70–79, and over 80. To increase the comparability among study years, we, respectively, combined the 18–19 and 20–29 data into one age category for the 2022 and 2023 survey data. Homeownership status was dichotomized into rent and own. Education status was categorized into six categories ranging from “less than high school diploma” to “post graduate work/degree or professional degree”. Gender was categorized into male, female, and third gender/other.

Within the 2021 and 2022 surveys, total household annual income (before taxes) was grouped into income categories of less than USD 10,000, USD 10,000 to USD 19,999, USD 20,000 to USD 29,999, USD 30,000 to USD 39,999, USD 40,000 to USD 49,999, USD 50,000 to USD 59,999, USD 60,000 to USD 99,999, USD 100,000 to USD 149,999, and USD 150,000 or more. However, in the 2023 NHS, FEMA altered their total household annual income (before taxes) grouping to include less than USD 10,000, USD 10,000 to USD 14,999, USD 15,000 to USD 24,999, USD 25,000 to USD 34,999, USD 35,000 to USD 49,999, USD 50,000 to USD 74,999, USD 75,000 to USD 99,999, USD 100,000 to USD 149,999, USD 150,000 to USD 199,999, and USD 200,000 or more. Due to the alternative categorization of the 2023 total household annual income, we chose to keep the respective income groupings instead of consolidating the groupings as reflected in Table 1. Census region was categorized into West, Midwest, Northeast, South, and Territories.

### 3.4. Statistical Analysis

Prior to conducting a statistical analysis of the three cleaned datasets, each dataset was inspected for multicollinearity among the included independent variables via the use of correlation matrices. The correlation matrix for each of the NHS survey years did not indicate any strong (0.70–0.89) or extremely correlated covariates as there were only five instances where the independent variables were in the 0.40–0.49 range of moderate correlation (0.40–0.69) [40]. Given the results of the correlation matrices, we moved forward with the analysis due to the low risk of multicollinearity among the independent variables analyzed in this study.

Descriptive statistics were generated to provide an overarching view of the effect the independent covariates had on individual-level pandemic preparedness behavior over the course of the three study years. Due to the dichotomous nature of the primary outcome, weighted univariate logistic regression was utilized as the main statistical analysis method. An interaction term was created between disaster preparedness status and census region due to the possibility of individuals in certain regions of the U.S. being more or less likely to prepare for a pandemic. This interaction term was specifically utilized to assess whether these two covariates, when taken together, influenced the odds of being prepared for a pandemic. Following the univariate analysis of each covariate (model 1), all of the significant covariates (including the interaction term) for the respective NHS survey year were included in a weighted multivariable logistic regression (model 2). To be able to compare the regression results from 2021–2023 under the same covariate conditions, we conducted a final weighted multivariate logistic regression (model 3) utilizing only the covariates that are consistently significant across all three study years. All analyses were conducted utilizing the statistical software Stata 18.0 (StataCorp, College Stations, TX, USA).

## 4. Results

### 4.1. Weighted Descriptive Statistics

The key results of the weighted descriptive statistical analysis related to disaster preparedness actions and the four FEMA influencers were summarized in Table 1 (see detailed Table S1 in the Supplemental Materials). The respondents included in the 2021, 2022, and 2023 weighted study samples consisted mostly of male and female respondents between the ages of 18 and 69 with the highest percentage of participants being 18–29 years old (Table S1). Interestingly, each of the three study years experienced the trend of more than half of the male participants being prepared for a pandemic, while over half of the females in each sample were unprepared for a pandemic. Within the 2021–2023 study samples, most of the participants resided in the West (2021: 37.2%; 2022: 31.2%; 2023: 23.5%) and South census regions (2021: 28.4%; 2022: 30.2%; 2023: 36.6%). Of those who were prepared for a pandemic, most of these individuals resided in the West census region in 2021 and 2022, while in 2023, the most individuals prepared for a pandemic were from the South census region. This result may be attributed to the population distribution in these areas.

**Figure 1:**
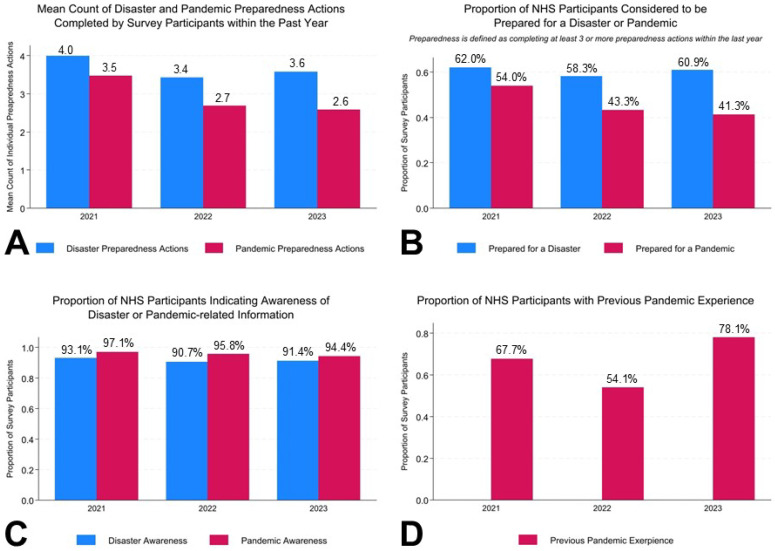
A visualization of the key descriptive statistics related to the level of individual disaster and pandemic preparedness among FEMA’s NHS participants from 2021 to 2023. (**A**) The mean count of disaster and pandemic preparedness actions completed by survey participants within the past year. (**B**) The proportion of NHS participants considered to be prepared for a disaster or pandemic by completing at least three or more preparedness actions within the last year. (**C**) The proportion of NHS participants that indicated an awareness of disaster or pandemic-related information by being aware of one or more of the provided preparedness topics. (**D**) The proportion of NHS participants with previous pandemic experience.

Over the course of the three study years, individuals tended to complete more disaster preparedness actions within the past year compared to pandemic preparedness actions (Figure 1A). In 2021, the average count of individual preparedness actions was 4.0 disaster preparedness actions and 3.5 pandemic preparedness actions. The mean count for both disaster and pandemic preparedness actions were highest in 2021 and decreased in 2022 to an average of 3.4 disaster preparedness actions and 2.7 pandemic preparedness actions. Comparatively, in 2023, the number of disaster actions among the study sample increased to 3.6 actions, but the number of pandemic preparedness actions continued to decrease to 2.6 actions. In 2021, 62.0% of individuals were prepared for a disaster, while only 58.3% and 60.9% of individuals were prepared for a disaster in 2022 and 2023, respectively (Figure 1B). The percentage of individuals prepared for a pandemic steadily decreased from 54.0% in 2021 to 43.3% in 2022 and 41.3% in 2023. Most individuals who prepared for a pandemic were coincidingly prepared for a disaster. In 2021, 90.6% of pandemic-prepared individuals were concurrently prepared for a disaster. However, this percentage was lowered in subsequent study years with 86.5% (2022) and 87.0% (2023) of pandemic-prepared individuals also being prepared for a disaster.

In survey year 2021, the top three disaster preparedness actions of “make your home safer”, “assemble or update supplies”, and “save for a rainy day” were the same as the top pandemic preparedness actions completed by survey participants (Table 2). However, in 2022, the top disaster and pandemic preparedness actions completed by participants in the last year both included “sign up for alerts and warnings” and “save for a rainy day”. The 2022 NHS participants opted to select “make your home safer” as the top pandemic preparedness action, but they did not select this action as frequently when classified as a disaster preparedness action. Among the 2023 study sample, the three preparedness actions with the highest proportion of completion within the last year were the same for both disaster and pandemic preparedness: “assemble or update supplies”, “make my home safer”, and “make a plan”. Early in the COVID-19 pandemic participants seemed to prioritize making their home safer, and nearing the end of the pandemic, participants participated in pandemic preparedness actions to prepare them for the next pandemic. Additionally, there was an observed overlap between preparedness actions selected to indicate disaster and pandemic preparedness within the last year. Among the weighted samples, less than a quarter of the participants selected identical preparedness actions for the questions assessing disaster and pandemic preparedness behavior within the last year (2021: 24.9%; 2022: 16.6%; 2023: 15.6%).

As seen in Table 1 and aspects of Figure 1, a large number of respondents from the included survey years reported having at least one or more of FEMA’s preparedness influencers. The proportion of study participants indicating awareness of pandemic-related information was slightly higher over the three survey years compared to participant’s awareness of disaster information (Figure 1C). The proportion of participants reporting awareness of at least one source of pandemic preparedness information decreased from 2021 to 2023 (2021: 97.1%; 2022: 95.8%; 2023: 94.4%). As seen in Table 2, the top preparedness subjects widely varied from disaster awareness to pandemic awareness, most likely due to the differing responses provided to participants for these questions. Early on in the pandemic, most respondents were aware of how to protect themselves or their family, followed by being aware of information on vaccines and avoiding infection. In 2022, respondents shifted away from prioritizing information about avoiding infection and were more aware of vaccine-related information, which coincides with the national rollout of COVID-19 vaccinations in 2021 and 2022. However, among 2023 NHS respondents, the overall awareness of pandemic preparedness information decreased, and most respondents were aware of information on avoiding COVID-19 infection. Awareness of disaster information remained consistent among survey participants from 2021 to 2023 with “making a plan” being the top disaster preparedness topic participants were aware of. As seen in Figure 1D, the proportion of participants demonstrating the FEMA preparedness influencer of previous pandemic experience fluctuated from 2021 to 2023 (2021: 67.7%; 2022: 54.1%; 2023: 78.1%).

In addition to the two previously mentioned preparedness influencers of previous experience and awareness of preparedness information, the NHS collected data on the influencers of self-efficacy and risk perception. Well over 80% of the respondents for each study year reported their confidence/self-efficacy for pandemic preparedness as “somewhat confident”, “moderately confident”, or “extremely confident” (2021: 84.9%; 2022: 89.7%; 2023: 90.9%). Over the three survey years, a greater proportion of individuals were unprepared for a pandemic for all confidence levels except for the moderately confident and extremely confident categories for all three study years. Among participants reporting extreme confidence in their ability to prepare for a pandemic, most of these individuals were also considered prepared for a pandemic. At least 80% of respondents reported their pandemic risk perception as “likely” or “very likely”. Overall, for the four preparedness influencers, there were observed differences in the percentage of responses based on stratification by pandemic preparedness status.

### 4.2. Logistic Regression Analysis

The full results of the three logistic regression models are included in Table S2 within the Supplemental Materials. The key predictors of individual-level pandemic preparedness pertaining to disaster preparedness status and the four influencers of pandemic preparedness are included in Table 3. According to both the univariate analysis and the adjusted multivariate analyses (Table 3) for the survey years between 2021 and 2023, there was a highly significant association at the 0.05 significance level between being prepared for a disaster and being prepared for a pandemic (*p*-values < 0.001). The univariate and multivariate associations between disaster preparedness and the likelihood of also being prepared for a pandemic was highest in 2021 and sharply decreased in 2022 and 2023. The multivariate results utilizing significant covariates across all three study years (model 3) found those who were prepared for a disaster were 21.35 (standard error (SE): 2.59, *p* < 0.001), 9.26 (SE: 0.87, *p* < 0.001), and 6.75 (SE: 0.59, *p* < 0.001) times more likely to be prepared for a pandemic in 2021, 2022, and 2023, respectively.

The univariate analysis (model 1) of the 2021 NHS data resulted in 12 covariates with at least one of the levels of the covariate being significant at α=0.05. The covariate of homeownership was removed for the multivariate analysis of the significant covariates for the 2021 NHS survey dataset. Among the 2022 univariate analysis results (model 1), 10 of the covariates contained at least one level with a significant result. The 2022 univariate analysis resulted in the non-statistically significant results for the covariates of homeownership, income level, and census region. However, the census region was included in the following multivariate regression due to the intention of exploring the interaction term between disaster preparedness and the census region in model 2. The univariate regression of the 2023 NHS dataset resulted in 12 significant covariates (model 1) with only the covariate of homeownership being removed prior to conducting multivariate analysis. Overall, all covariates related to the four preparedness influencers had highly statistically significant results (*p* < 0.001) in the univariate analysis of pandemic preparedness; thus, suggesting that FEMA’s four influencers of disaster preparedness can be applied to influence an individual’s pandemic preparedness behaviors.

In the multivariate logistic regression of the significant terms for the 2021 NHS (model 2), individuals who participated in at least three disaster preparedness actions in the last year were 17.97 (SE: 4.55, *p* < 0.001) more likely to have completed at least three pandemic preparedness actions in the last year (prepared for a pandemic). The multivariate logistic regression with the investigation of an interaction term for the 2022 data resulted in individuals completing at least three disaster preparedness activities being 11.69 times more likely to be prepared for a pandemic (SE: 2.42, *p* < 0.001). In 2023, the multivariate logistic regression of all significant covariates for the 2023 NHS individuals who were prepared for a disaster were 9.16 times more likely to be prepared for a pandemic (SE: 1.83, *p* < 0.001). Overall, the multivariate regression of significant covariates tailored to each study year resulted in the interaction term being statistically insignificant; thus, we deemed the interaction term as unnecessary for the proceeding model.

**Figure 2:**
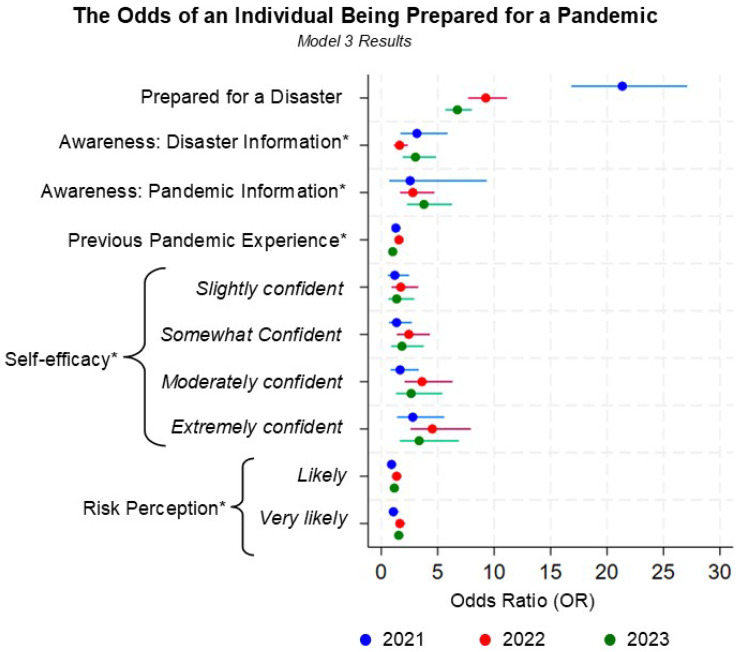
A forest plot of key predictors of personal pandemic preparedness identified via weighted multivariate logistic regressions using covariates that were consistently significant for all three study years (model 3). This figure only contains key covariates from model 3. Additional covariates in model 3 include race, age, educational status, and gender. * Denotes FEMA’s four “influencers” of personal preparedness.

To make a comparison between the included NHS survey years, the third logistic model includes covariates identified as significant across the three datasets. Across all three study years, the significant covariates (model 3) include disaster preparedness, awareness of disaster information, awareness of pandemic information, previous pandemic experience, pandemic self-efficacy, pandemic perception, race, age, education category, and gender. The key results from model 3 are depicted in Figure 2 for variables pertaining to disaster preparedness and FEMA’s four influencers of pandemic preparedness. As expected, the significant relationship between individual-level disaster preparedness and pandemic preparedness was strongest in 2021 as most respondents took preparedness actions during the initial year of the pandemic (OR: 21.35, SE: 2.59, *p* < 0.001) when adjusting for the other nine covariates. In 2022, the adjusted relationship between disaster and pandemic preparedness was reduced to 9.26 (SE: 0.87, *p* < 0.001). This rapid decrease could be attributed to preparedness status being determined by the participants actions within the last year; thus, respondents’ preparedness actions might have rapidly decreased as the world reopened and individuals became normalized to the effects of the pandemic. The adjusted odds ratio settled to 6.75 in 2023 (SE: 0.59, *p* < 0.001).

Across all three study years, the adjusted multivariate model using consistent significant covariates resulted in highly statistically significant results for awareness of disaster preparedness information and the extremely confident level of pandemic confidence/self-efficacy. Under adjustment, those aware of disaster preparedness information were 3.16, 1.61, and 3.04 times as likely to be prepared for a pandemic in 2021, 2022, and 2023 compared to those unaware of at least one source of disaster information (2021: SE: 1.00, *p* < 0.001; 2022: SE: 0.30, *p* < 0.01; 2023: SE: 0.73, *p* < 0.001). Individuals who were extremely confident in their ability to prepare for a pandemic were 2.79, 4.52, 3.36 times more likely to be prepared for a pandemic when adjusting for all other covariates (2021: SE: 0.99, *p* < 0.01; 2022: SE: 1.30, *p* < 0.001; 2023: SE: 1.23, *p* < 0.001). The 2021 and 2023 dataset demonstrated high statistically significant relationship between pandemic risk perception of “very likely” and exhibiting pandemic preparedness behavior. Previous pandemic experience had a statistically significant relationship with pandemic preparedness among the 2021 and 2022 dataset. Interestingly, the covariate for awareness of pandemic preparedness information only exhibited a statistically significant relationship in 2022 and 2023, which suggests the effect of preparedness information on the likeliness to prepare for a pandemic increased over the span of the COVID-19 pandemic (2022: OR: 2.80, SE: 0.74, *p* < 0.001; 2023: OR: 3.78, SE: 0.98, *p* < 0.001).

## 5. Discussion

Through the use of nationally representative data, the results of this study effectively demonstrate the highly statistically significant relationship between individual-level disaster preparedness and pandemic preparedness behavior among the U.S. adult population. Historically, disaster preparedness and pandemic preparedness efforts have been seen as siloed processes, especially at the individual-level. Prior to the COVID-19 pandemic, individual-level pandemic preparedness among Americans was not emphasized or widely advertised among federal agencies; therefore, most adults in the U.S. likely did not actively prepare for a pandemic in addition to their routine disaster preparedness efforts. The results from this study demonstrate that the individual-level completion of at least three disaster preparedness actions in the last year will increase their likeliness to be prepared for a pandemic, and this relationship also holds for pandemic preparedness influencing a likeliness to be prepared for a disaster. Furthermore, the investment in an overall increase in both pandemic and disaster preparedness at the individual-level, through a rise in educational programs and preparedness campaigns, can increase overall community preparedness and indirectly impact resilience to disasters whether they are natural or biological hazards. These results support enhanced collaboration between public health and emergency management to increase individual preparedness.

The logistic regression analysis further identified key predictors of individual-level pandemic preparedness among U.S. adults with emphasis on the influence of FEMA’s preparedness influencers on pandemic preparedness. Prior to this study, no study has investigated the application of FEMA’s four preparedness influencers on an individual’s likeliness to prepare for a pandemic over the course of multiple study years. This work expands on the initial progress made by Chen et al. in 2024 by demonstrating the consistent role the four FEMA influencers have on the likelihood of an individual being prepared for a pandemic over the course of three NHS study years [19]. The univariate logistic regression results highlight the transferable use of FEMA’s disaster preparedness influencers as predictors of pandemic preparedness. Thus, public health departments and agencies can leverage these influencers to guide their outreach and education of community members to ultimately increase individual-level pandemic preparedness. Additionally, these results could generate interest in further investigation by public health agencies and entities into the application of FEMA’s preparedness methods to public health preparedness, and to decern the addition of predictors specifically tailored to public health.

Similarly to the influence that being prepared for disaster has on the likelihood of being prepared for a pandemic, the awareness of disaster preparedness information also positively impacts the likelihood of individual-level pandemic preparedness across all three NHS study years. The influence of the awareness of disaster- and pandemic-related information highlights the importance of investing in accessible and actionable information as it positively influences preparedness actions. The descriptive statistical results of the awareness of disaster and pandemic information demonstrate the varying informational needs for disaster versus pandemic preparedness. This highlights the need for further investigation into the information and media that individuals seek in a disaster versus a pandemic, and how this influences individual preparedness actions.

As seen in the descriptive statistic results, the trends in taking preparedness actions and being aware of event-related information varied with the time since the start of the COVID-19 pandemic in 2020. With the increase in time, the average number of pandemic preparedness actions, along with the percentage of participants aware of pandemic-related information, decreased from 2021 to 2023. This decline could possibly be attributed to society’s return to everyday activities, shifts in the economy, information and decision fatigue from the pandemic, alterations in public messaging, and the evolution of the pandemic response. However, the trend in disaster preparedness activities and awareness of disaster information decreased from 2021 to 2022 followed by an increase in 2023, which could be attributed to the increase in natural disasters in addition to other factors. Overall, the trends in the descriptive results demonstrate the investment in increasing pandemic preparedness is most effective in increasing pandemic preparedness activities in the early years of a pandemic when community response capacity may be low, and the societal disruption of the event is high; thus, this finding emphasizes the need to increase the availability of individual pandemic preparedness online resources, community engagement activities, trainings, and educational campaigns prior to onset of the next pandemic. Furthermore, the key predictors of pandemic preparedness identified in this study, including FEMA’s preparedness influencers, should be utilized to inform the development and delivery of pandemic preparedness resources and campaigns to enhance the potential impact of these efforts and further normalize a culture of pandemic preparedness in the U.S.

### Limitations

Despite the important impacts of this study on bolstering individual pandemic and disaster preparedness in the U.S., the study has limitations that could affect the impact of these results. The analysis utilized in the study was directly limited by the nature of the nationally representative data. Although the data are nationally representative, the dataset and the accompanying models do not include all of the potential confounding variables affecting an individual’s decision to take preparedness actions for future pandemics and disasters; thus, the resulting models, like many statistical models, may have lingering limitations that impact the ability to fully assess the association of interest.

Furthermore, in this analysis, preparedness status was determined by the threshold of completing at least three preparedness actions within the last year. Despite the use of this metric in a previous analysis and FEMA’s summary reports, this assumption may not be applicable in all situations. The NHS assumed that the same preparedness actions utilized for a disaster are the same actions needed to prepare for a pandemic, which could contribute to the possibly exaggerated relationship between disaster preparedness and pandemic preparedness seen in model 3. Due to this assumption, this study’s results do not consider additional preparedness actions needed to accurately prepare for a pandemic that are not included in the actions needed to prepare for a disaster. For future NHSs, FEMA should collaborate with federal public health agencies, such as the CDC, to confirm that these actions are appropriate for individual pandemic preparedness and add actions as deemed necessary to ensure that the options respondents can select from are comprehensive. Similarly, the NHS supplied respondents with two differing lists of options for indicating their awareness of disaster and pandemic information, which limited the ability for the study’s analysis to compare the respondents’ awareness of information for a disaster compared to a pandemic.

Although the exploration of the FEMA influencers was a benefit of this study, the FEMA preparedness influencers were created specifically for predicting disaster preparedness behaviors; thus, the study’s analysis was limited to only considering the influencers of individual disaster preparedness behavior. In the future, FEMA should consider collaborating with federal public health agencies to further investigate the application of these indicators to individual pandemic preparedness behaviors and amend these predictors as needed. Although the study has limitations, the results demonstrate the synergistic influence of individual-level disaster and pandemic preparedness behaviors, which can be capitalized to bolster community resilience and mitigate the impacts of future natural and biological events.

Given the invaluable information that the annual NHS provides, it is imperative that FEMA’s NHS continues to be conducted. It is also crucial for this data to remain publicly accessible for states, counties, and the academic community to utilize to enhance preparedness efforts and make evidence-based decisions. In light of the current federal funding and programmatic uncertainties, state and county public health and emergency management agencies should consider jointly conducting their own household survey to assess personal preparedness behaviors among their residents to better inform their preparedness campaigns and activities in the future.

## 6. Conclusions

The statistically significant results of the final multivariate logistic models of the nationally representative NHS data from 2021 to 2023 demonstrate the large, positive relationship between an individual being prepared for a disaster and their odds of concurrently being prepared for a pandemic. The results of this study support enhanced future collaboration between public health and emergency management entities to increase individual-level preparedness regardless of the event type as an increase in individual-level preparedness actions for one field enhances the odds of preparedness in the other. Practically, the results from this study support the need for enhanced community engagement to increase pandemic preparedness actions prior to a pandemic, cross-field collaboration to bolster personal preparedness to all hazards, and the use of governmental policies and public communication programs to incentivize personal preparedness in the U.S. Overall, the study results demonstrate the need for public health and emergency management entities at all levels of government to collaborate in supporting the development of evidence-based programs and resources to increase individual preparedness for both disaster and pandemics to bolster preparedness and community resilience nationwide.

## Figures and Tables

**Table 1:** Key weighted sample characteristics among the sample of 2021–2023 FEMA national household survey participants stratified by individual pandemic preparedness status ^a^.

Survey Year	2021			2022			2023		
	Unprepared	Prepared	Total	Unprepared	Prepared	Total	Unprepared	Prepared	Total
N	2907 (46.0%)	3414 (54.0%)	6321	3498 (56.7%)	2673 (43.3%)	6171	3757 (58.7%)	2647 (41.3%)	6404
At Least 3 Disaster Preparedness Actions in the Past Year ^b^									
Unprepared	2082 (71.6%)	319 (9.4%)	2401 (38.0%)	2215 (63.3%)	360 (13.5%)	2576 (41.7%)	2157 (57.4%)	345 (13.0%)	2502 (39.1%)
Prepared	825 (28.4%)	3095 (90.6%)	3920 (62.0%)	1283 (36.7%)	2313 (86.5%)	3596 (58.3%)	1599 (42.6%)	2303 (87.0%)	3902 (60.9%)
Awareness of Disaster Preparedness Information									
No	377 (13.0%)	57 (1.7%)	434 (6.9%)	503 (14.4%)	72 (2.7%)	575 (9.3%)	507 (13.5%)	42 (1.6%)	549 (8.6%)
Yes	2530 (87.0%)	3357 (98.3%)	5887 (93.1%)	2995 (85.6%)	2601 (97.3%)	5597 (90.7%)	3250 (86.5%)	2605 (98.4%)	5855 (91.4%)
Awareness of Pandemic Preparedness Information									
No	161 (5.5%)	23 (0.7%)	183 (2.9%)	235 (6.7%)	27 (1.0%)	262 (4.2%)	331 (8.8%)	31 (1.2%)	362 (5.6%)
Yes	2746 (94.5%)	3392 (99.3%)	6138 (97.1%)	3263 (93.3%)	2647 (99.0%)	5909 (95.8%)	3426 (91.2%)	2616 (98.8%)	6042 (94.4%)
Previous Pandemic Experience									
No	1120 (38.8%)	912 (26.8%)	2033 (32.3%)	1826 (52.2%)	1009 (37.7%)	2835 (45.9%)	912 (24.3%)	493 (18.6%)	1406 (21.9%)
Yes	1768 (61.2%)	2496 (73.2%)	4264 (67.7%)	1672 (47.8%)	1665 (62.3%)	3337 (54.1%)	2844 (75.7%)	2154 (81.4%)	4998 (78.1%)
Pandemic Confidence/Self-efficacy									
Not at all confident	137 (4.7%)	50 (1.5%)	186 (2.9%)	193 (5.5%)	26 (1.0%)	218 (3.5%)	152 (4.0%)	20 (0.8%)	172 (2.7%)
Slightly confident	465 (16.0%)	304 (8.9%)	769 (12.2%)	320 (9.1%)	94 (3.5%)	414 (6.7%)	335 (8.9%)	76 (2.9%)	411 (6.4%)
Somewhat confident	905 (31.1%)	721 (21.1%)	1626 (25.7%)	1025 (29.3%)	484 (18.1%)	1509 (24.4%)	981 (26.1%)	387 (14.6%)	1368 (21.4%)
Moderately confident	883 (30.4%)	1209 (35.4%)	2092 (33.1%)	1140 (32.6%)	997 (37.3%)	2136 (34.6%)	1271 (33.8%)	979 (37.0%)	2250 (35.1%)
Extremely confident	517 (17.8%)	1132 (33.1%)	1649 (26.1%)	820 (23.5%)	1073 (40.1%)	1894 (30.7%)	1019 (27.1%)	1184 (44.7%)	2203 (34.4%)
Pandemic Risk Perception									
Unlikely	439 (15.1%)	335 (9.8%)	773 (12.2%)	633 (18.1%)	289 (10.8%)	922 (14.9%)	765 (20.4%)	330 (12.4%)	1094 (17.1%)
Likely	1285 (44.2%)	1223 (35.8%)	2508 (39.7%)	1522 (43.5%)	1006 (37.6%)	2528 (41.0%)	1755 (46.7%)	1069 (40.4%)	2824 (44.1%)
Very likely	1184 (40.7%)	1856 (54.4%)	3040 (48.1%)	1344 (38.4%)	1379 (51.6%)	2722 (44.1%)	1237 (32.9%)	1249 (47.2%)	2485 (38.8%)

Key characteristics of the study samples are summarized in this table. This table highlights the sample statistics of each sample from the 2021 to 2023 NHS pertaining to the covariates of high interest: disaster preparedness actions and the four FEMA influencers of preparedness behavior. See Table S1 for the full details of the characteristics of the study samples. The table above was weighted using FEMA’s geography-based weighting scheme. For all categorical variables included in the table above, the summary statistics are displayed in the n (%) format. Throughout the table, within group percentages might add up to slightly more than 100 due to rounding. ^a^ Level of pandemic preparedness was categorized into unprepared (an individual achieved less than 3 pandemic preparedness activities within the past year) and prepared (an individual has achieved 3 or more pandemic preparedness activities within the past year). ^b^ Level of disaster preparedness was categorized into unprepared (an individual has achieved less than 3 disaster preparedness activities within the past year) and prepared (an individual has achieved 3 or more disaster preparedness activities within the past year).

**Table 2:** Preparedness actions and awareness topics with the highest proportion of completion or recognition by NHS respondents from 2021 to 2023.

	**Top Disaster Preparedness Actions ** **(% of Respondents)**			**Top Pandemic Preparedness Actions ** **(% of Respondents)**		
2021	Make your home safer (46.8%)	Assemble or update supplies (46.7%)	Save for a rainy day (45.7%)	Make your home safer (46.1%)	Assemble or update supplies (43.4%)	Save for a rainy day (41.3%)
2022	Sign up for alerts and warnings (47.8%)	Make a plan (42.6%)	Save for a rainy day (37.8%)	Make your home safer (34.1%)	Save for a rainy day (32.3%)	Sign up for alerts and warnings (30.4%)
2023	Assemble or update supplies (50.0%)	Make a plan (39.3%)	Make your home safer (37.9%)	Assemble or update supplies (38.1%)	Make your home safer (31.4%)	Make a plan (28.1%)
	**Top Disaster Awareness Subjects** **(% of Respondents)**			**Top Pandemic Awareness Subjects** **(% of Respondents)**		
2021	Make a plan (57.7%)	Sign up for alerts and warnings (53.7%)	Save for a rainy day (51.8%)	Protecting yourself/family (74.3%)	Vaccines (72.4%)	Avoiding infection (70.0%)
2022	Make a plan (53.0%)	Sign up for alerts and warnings (50.1%)	Save for a rainy day (44.8%)	Vaccines (75.3%)	Protecting yourself/family (66.3%)	Testing (65.4%)
2023	Make a plan (57.6%)	Assemble or update supplies (52.8%)	Know your evacuation routes (51.2%)	Avoiding infection (63.5%)	Protecting yourself/family (60.8%)	Testing (60.6%)

**Table 3:** Key predictors of individual-level pandemic preparedness via weighted univariate and multivariate logistic regressions ^a^.

Survey Year	2021			2022			2023		
Regression Type ^b^	Model 1 OR(SE)	Model 2 OR(SE)	Model 3 OR(SE)	Model 1 OR(SE)	Model 2 OR(SE)	Model 3 OR(SE)	Model 1 OR(SE)	Model 2 OR(SE)	Model 3 OR(SE)
Prepared for a Disaster ^c^ (*Ref: No*)	24.46 *** (2.85)	17.97 *** (4.55)	21.35 *** (2.59)	11.08 *** (0.96)	11.69 *** (2.42)	9.26 *** (0.87)	9.01 *** (0.74)	9.16 *** (1.83)	6.75 *** (0.59)
Awareness of Disaster Preparedness Information (*Ref: No*)	8.78 *** (2.19)	3.06 *** (0.97)	3.16 *** (1.00)	6.06 *** (1.00)	1.61 * (0.30)	1.62 ** (0.30)	9.58 *** (2.13)	3.02 *** (0.74)	3.04 *** (0.73)
Awareness of Pandemic Preparedness Information (*Ref: No*)	8.80 *** (3.82)	2.67 (1.78)	2.56 (1.69)	7.17 *** (1.62)	2.79 *** (0.73)	2.80 *** (0.74)	8.10 *** (1.84)	3.79 *** (0.98)	3.78 *** (0.98)
Previous Pandemic Experience (*Ref: No*)	1.73 *** (0.15)	1.28 * (0.15)	1.29 * (0.15)	1.80 *** (0.12)	1.55 *** (0.13)	1.56 *** (0.13)	1.40 *** (0.11)	1.01 (0.10)	1.01 (0.10)
Pandemic Confidence/Self-efficacy (*Ref: Not at all confident*)									
Slightly confident	1.80 (0.55)	1.22 (0.44)	1.20 (0.44)	2.20 ** (0.61)	1.71 (0.56)	1.73 (0.57)	1.69 (0.55)	1.37 (0.54)	1.36 (0.53)
Somewhat confident	2.20 ** (0.64)	1.38 (0.48)	1.36 (0.48)	3.53 *** (0.89)	2.42 ** (0.70)	2.44 ** (0.71)	2.93 *** (0.89)	1.84 (0.68)	1.83 (0.68)
Moderately confident	3.78 *** (1.09)	1.74 (0.59)	1.67 (0.58)	6.54 *** (1.61)	3.56 *** (1.02)	3.61 *** (1.03)	5.72 *** (1.71)	2.68 ** (0.98)	2.65 ** (0.97)
Extremely confident	6.04 *** (1.76)	2.87 ** (0.99)	2.79 ** (0.99)	9.79 *** (2.41)	4.49 *** (1.29)	4.52 *** (1.30)	8.63 *** (2.58)	3.41 *** (1.25)	3.36 *** (1.23)
Pandemic Risk Perception (*Ref: Unlikely*)									
Likely	1.25 (0.17)	0.92 (0.17)	0.91 (0.17)	1.45 *** (0.16)	1.37 * (0.18)	1.36 * (0.18)	1.41 *** (0.13)	1.18 (0.14)	1.16 (0.14)
Very likely	2.06 *** (0.28)	1.06 (0.20)	1.08 (0.20)	2.25 *** (0.24)	1.65 *** (0.21)	1.65 *** (0.21)	2.34 *** (0.22)	1.53 *** (0.19)	1.55 *** (0.19)
Intercept		0.01 *** (0.01)	0.01 *** (0.01)		0.01 *** (0.00)	0.01 *** (0.00)		0.01 *** (0.00)	0.01 *** (0.00)

*** *p* < 0.001, ** *p* < 0.01, * *p* < 0.05. Key predictors of personal pandemic preparedness identified via weighted univariate and multivariate logistic regressions are summarized in this table. This table highlights the weighted logistic regression results from three models pertaining to the covariates of high interest: disaster preparedness actions and the four FEMA influencers of preparedness behavior. However, additional covariates utilized in this analysis include race, age, homeownership, education status, gender, total household annual income, census region, and an interaction term between disaster preparedness and census region. See Table S2 for the full details of the logistic regression analysis. The table above was weighted using FEMA’s geography-based weighting scheme. ^a^ The level of pandemic preparedness was categorized into unprepared (an individual has achieved less than 3 pandemic preparedness activities within the past year) and prepared (an individual has achieved 3 or more pandemic preparedness activities within the past year). ^b^ For each survey year, regression type is defined as univariate logistic regression (model 1), multivariate logistic regression of significant covariates unique to each survey year (model 2), and multivariate logistic regression of significant covariates across all three survey years (model 3). ^c^ Level of disaster preparedness was categorized into unprepared (an individual has achieved less than 3 disaster preparedness activities within the past year) and prepared (an individual has achieved 3 or more disaster preparedness activities within the past year).

## Data Availability

The STATA code utilized for this analysis is included as a Supplemental Material. The data utilized in this study are publicly available on FEMA’s website at: https://www.fema.gov/about/openfema/data-sets/national-household-survey (accessed on 3 December 2023).

## Supplementary Materials

The following supporting information can be downloaded at: https://www.mdpi.com/article/10.3390/ijerph22050702/s1, Table S1: The weighted sample characteristics of 2021–2023 FEMA national household survey participants stratified by individual pandemic preparedness status; Table S2: Predictors of individual-level pandemic preparedness via weighted univariate and multivariate logistic regressions. Code: STATA code utilized for the analysis of FEMA’s NHS from 2021 to 2023.

## Abbreviations

The following abbreviations are used in this manuscript:

CDC	Centers for Disease Control and Prevention
DHS	Department of Homeland Security
FEMA	Federal Emergency Management Agency
HHS	Department of Health and Human Services
NHS	National Household Survey
OR	Odds Ratio
PHEP	Public Health Emergency Preparedness
SE	Standard Error
