# Supplementary material for: The Positive Influence of Individual-Level Disaster Preparedness on the Odds of Individual-Level Pandemic Preparedness—Insights from FEMA’s 2021–2023 National Household Survey
Source: Int J Environ Res Public Health. 2025 Apr 29;22(5):702. doi: 10.3390/ijerph22050702 (PMC12111411; doi:10.3390/ijerph22050702)
Supplement: Supplementary file 1 [file ijerph-22-00702-s001.zip › ijerph-3522834_SuplementalMaterials_STATA_Code_04262025.pdf]

```
*STATA Code Utilized for the Analysis of FEMA's NHS from 2021-2023*

*-------------------------------------------------------------------

****READ ME****

*This is a Stata .do file (compatiable with PCs) corresponding to the analyses of the Federal Emergency Management Agency's (FEMA) National Household Survey datasets from 2021-2023 for the article titled The Positive Influence of Individual-level Disaster Preparedness on the Odds of Individual-level Pandemic Preparedness – Insights from FEMA's 2021-2023 National Household Survey (authored by D. Mitcham and C. Watson)*

*To use this .do file on a MAC please convert all of the "\" to "/" and remove the "C:\" utilized throughout this file*

*Questions regarding the following .do file should be sent to the corresponding author, D. Mitcham (dmitcham@jhu.edu)*

*-------------------------------------------------------------------

****STATA Code****

*Set working directory and start a log file*
cd "C:\Location"
log using "C:\Location\FEMA_NHS_2021_2023_LogFile.log", replace

***2021 NHS Dataset***
tempfile temp_data
import excel "C:\Location\fema_national_household_survey_data_and_codebook_2021.xlsx", sheet("2021 NHS General Data") firstrow clear
export delimited using `temp_data', replace
import delimited `temp_data', varnames(2) clear

save "C:\Location\fema_national_household_survey_data_and_codebook_2021.dta", replace

*Number of Observations Prior to Data Cleaning*
count

*Disaster Data*

*Awareness of Disaster Preparedness Information - 2021*

foreach var of varlist a3_a a3_b a3_c a3_d a3_e a3_f a3_g a3_h a3_i a3_j a3_k a3_l {
    replace `var' = "1" if `var' != ""
}

foreach var of varlist a3_a a3_b a3_c a3_d a3_e a3_f a3_g a3_h a3_i a3_j a3_k a3_l a3_m a3_n a3_o{
    replace `var' = "0" if `var' == ""
}
replace a3_m = "0" if a3_m=="None"
drop if strpos(a3_n, "Don't know")
drop if strpos(a3_o, "Prefer not to answer")
destring a3_a a3_b a3_c a3_d a3_e a3_f a3_g a3_h a3_i a3_j a3_k a3_l a3_m a3_n a3_o, replace
egen awareness_gen = rowtotal(a3_a a3_b a3_c a3_d a3_e a3_f a3_g a3_h a3_i a3_j a3_k a3_l)
tabulate awareness_gen
replace awareness_gen = 1 if awareness_gen>= 1
replace awareness_gen = 0 if awareness_gen< 1
label define awareness_gen_lab 0 "No" 1 "Yes"
label values awareness_gen awareness_gen_lab
label variable awareness_gen "Awareness of Disaster Preparedness Information"
tabulate awareness_gen
```

```
*Number of Disaster Preparedness Actions within the Last Year and At Least Three Disaster Preparedness Actions - 2021*
foreach var of varlist prepb_a prepb_b prepb_c prepb_d prepb_e prepb_f prepb_g prepb_h prepb_i prepb_j prepb_k prepb_l {
    replace `var' = "1" if `var' != ""
}

foreach var of varlist prepb_a prepb_b prepb_c prepb_d prepb_e prepb_f prepb_g prepb_h prepb_i prepb_j prepb_k prepb_l prepb_m prepb_n prepb_o{
    replace `var' = "0" if `var' == ""
}
replace prepb_m = "0" if prepb_m=="None"
drop if strpos(prepb_n, "Don't know")
drop if strpos(prepb_o, "Prefer not to answer")
destring prepb_a prepb_b prepb_c prepb_d prepb_e prepb_f prepb_g prepb_h prepb_i prepb_j prepb_k prepb_l prepb_m prepb_n prepb_o, replace
egen num_prep_action_general = rowtotal(prepb_a prepb_b prepb_c prepb_d prepb_e prepb_f prepb_g prepb_h prepb_i prepb_j prepb_k prepb_l)
label variable num_prep_action_general "Number of Disaster Preparedness Actions within the Last Year"
summarize num_prep_action_general
tabulate num_prep_action_general
gen atleast3_prep_action_gen = num_prep_action_general
replace atleast3_prep_action_gen = 0 if atleast3_prep_action_gen== 0 | atleast3_prep_action_gen== 1 | atleast3_prep_action_gen== 2
replace atleast3_prep_action_gen = 1 if inrange(atleast3_prep_action_gen, 3, 12)
label define atleast3_prep_action_gen_lab 0 "Unprepared" 1 "Prepared"
label values atleast3_prep_action_gen atleast3_prep_action_gen_lab
label variable atleast3_prep_action_gen "At Least 3 Disaster Preparedness Actions in the Past Year"
tabulate atleast3_prep_action_gen

*Outcome - Pandemic Preparedness Actions within the Last Year*

*Number of Pandemic Preparedness Actions within the Last Year and At Least Three Pandemic Preparedness Actions - 2021*
foreach var of varlist prepa_a prepa_b prepa_c prepa_d prepa_e prepa_f prepa_g prepa_h prepa_i prepa_j prepa_k prepa_l {
    replace `var' = "1" if `var' != ""
}

foreach var of varlist prepa_a prepa_b prepa_c prepa_d prepa_e prepa_f prepa_g prepa_h prepa_i prepa_j prepa_k prepa_l prepa_m prepa_n prepa_o{
    replace `var' = "0" if `var' == ""
}
replace prepa_m = "0" if prepa_m=="None"
drop if strpos(prepa_n, "Don't know")
drop if strpos(prepa_o, "Prefer not to answer")
destring prepa_a prepa_b prepa_c prepa_d prepa_e prepa_f prepa_g prepa_h prepa_i prepa_j prepa_k prepa_l prepa_m prepa_n prepa_o, replace
egen num_prep_action_pandemic = rowtotal(prepa_a prepa_b prepa_c prepa_d prepa_e prepa_f prepa_g prepa_h prepa_i prepa_j prepa_k prepa_l)
label variable num_prep_action_pandemic "Number of Pandemic Preparedness Actions within the Last Year"
summarize num_prep_action_pandemic
tabulate num_prep_action_pandemic
gen atleast3_prep_action_pan = num_prep_action_pandemic
replace atleast3_prep_action_pan = 0 if atleast3_prep_action_pan== 0 | atleast3_prep_action_pan== 1 | atleast3_prep_action_pan== 2
replace atleast3_prep_action_pan = 1 if inrange(atleast3_prep_action_pan, 3, 12)
label define atleast3_prep_action_pan_lab 0 "Unprepared" 1 "Prepared"
label values atleast3_prep_action_pan atleast3_prep_action_pan_lab
label variable atleast3_prep_action_pan "At Least 3 Pandemic Preparedness Actions in the Past Year"
tabulate atleast3_prep_action_pan

*FEMA's Four Influencers of Preparedness - Pandemics*
```

```

*Pandemic Risk Perception*
drop if strpos(l1a, "Don't know")
drop if strpos(l1a, "Prefer not to answer")
replace l1a = "0" if l1a=="Unlikely"
replace l1a = "1" if l1a=="Likely"
replace l1a = "2" if l1a=="Very likely"
replace l1a = "2" if l1a=="Very Likely"
destring l1a, replace
label define l1a_lab 0 "Unlikely" 1 "Likely" 2 "Very likely"
label values l1a l1a_lab
label variable l1a "Pandemic Risk Perception Q"


*Previous COVID-19 Pandemic Experience - Combined*
tabulate genexpa
gen genexpa_combined = ""
replace genexpa_combined = "Yes" if genexpa == "Yes" | genexpa_1 == "Yes"
replace genexpa_combined = "No" if (genexpa == "No" | genexpa_1 == "No") & genexpa_combined != "Yes"
replace genexpa_combined = "Don't Know" if (genexpa == "Don't Know" & genexpa_1 == "Don't Know") | (genexpa == "Prefer Not To Answer" & genexpa_1 == "Prefer Not To Answer") | (genexpa == "Don't Know" & genexpa_1 == "Prefer Not To Answer") | (genexpa == "Prefer Not To Answer" & genexpa_1 == "Don't Know") & genexpa_combined == ""
drop if strpos(genexpa_combined, "Don't know")
replace genexpa_combined ="0" if genexpa_combined=="No"
replace genexpa_combined ="1" if genexpa_combined=="Yes"
destring genexpa_combined, replace
label define genexpa_combined_lab 0 "No" 1 "Yes"
label values genexpa_combined genexpa_combined_lab
rename genexpa genexpa_0
rename genexpa_combined genexpa
label variable genexpa "Previous Pandemic Experience"
tabulate genexpa


*Pandemic Confidence/Self-efficacy*
drop if strpos(c2_1g, "Don't know")
drop if strpos(c2_1g, "Prefer not to answer")
replace c2_1g = "1" if c2_1g=="Not at all confident"
replace c2_1g = "2" if c2_1g=="Slightly confident"
replace c2_1g = "3" if c2_1g=="Somewhat confident"
replace c2_1g = "4" if c2_1g=="Moderately confident"
replace c2_1g = "5" if c2_1g=="Extremely confident"
destring c2_1g, replace
label define c2_1g_lab 1 "Not at all confident" 2 "Slightly confident" 3 "Somewhat confident" 4 "Moderately confident" 5 "Extremely confident"
label values c2_1g c2_1g_lab
label variable c2_1g "Pandemic Confidence/Self-efficacy"

*Awareness of Pandemic Preparedness Information - 2021*

foreach var of varlist a1_a a1_b a1_c a1_d a1_e a1_f a1_g a1_h{
    replace `var' = "1" if `var' != ""
}

foreach var of varlist a1_a a1_b a1_c a1_d a1_e a1_f a1_g a1_h a1_i a1_j a1_k{
    replace `var' = "0" if `var' == ""
}
replace a1_i = "0" if a1_i=="None"
drop if strpos(a1_j, "Don't know")
drop if strpos(a1_k, "Prefer not to answer")
destring a1_a a1_b a1_c a1_d a1_e a1_f a1_g a1_h a1_i a1_j a1_k, replace
egen awareness_pan = rowtotal(a1_a a1_b a1_c a1_d a1_e a1_f a1_g a1_h)
```

```
replace awareness_pan = 1 if awareness_pan>= 1
replace awareness_pan = 0 if awareness_pan< 1
label define awareness_pan_lab 0 "No" 1 "Yes"
label values awareness_pan awareness_pan_lab
label variable awareness_pan "Awareness of Pandemic Preparedness Information"

*Demographic Information*

*Census Region*
replace census_region ="1" if census_region=="West"
replace census_region ="2" if census_region=="Midwest"
replace census_region ="3" if census_region=="Northeast"
replace census_region ="4" if census_region=="South"
replace census_region ="5" if census_region==""
replace census_region ="5" if census_region=="N/A"
replace census_region ="5" if census_region=="Territories"
destring census_region, replace
label define census_region_lab 1 "West" 2 "Midwest" 3 "Northeast" 4 "South" 5 "Territories"
label values census_region census_region_lab
label variable census_region "Census Region"

*Age* 2021
replace age = "1" if age=="18-29"
replace age = "2" if age=="30-39"
replace age = "3" if age=="40-49"
replace age = "4" if age=="50-59"
replace age = "5" if age=="60-69"
replace age = "6" if age=="70-79"
replace age = "7" if age=="Over 80"
replace age = "7" if age=="80+"
destring age, replace
label define age_lab 1 "18-29" 2 "30-39" 3 "40-49" 4 "50-59" 5 "60-69" 6 "70-79" 7 "Over 80"
label values age age_lab
label variable age "Age"

*Gender - 2021*
replace gender = "0" if gender=="Male"
replace gender = "1" if gender=="Female"
replace gender = "2" if gender=="Third-Gender/Other"
replace gender = "2" if gender=="Third-gender/Other"
destring gender, replace
label define gender_lab 0 "Male" 1 "Female" 2 "Third Gender/Other"
label values gender gender_lab
label variable gender "Gender"

*Education*
replace educ = "0" if educ=="Less than high school diploma"
replace educ = "1" if educ=="High school degree or diploma"
replace educ = "2" if educ=="Some college, no degree"
replace educ = "3" if educ=="Associate's degree"
replace educ = "4" if educ=="Bachelor's degree"
replace educ = "5" if educ=="Post graduate work/degree or professional degree"
destring educ, replace
label define educ_lab 0 "Less than high school diploma" 1 "High school degree or diploma" 2 "Some
college, no degree" 3 "Associate's degree" 4 "Bachelor's degree" 5 "Post graduate work/degree or
professional degree"
label values educ educ_lab
label variable educ "Education"

*Ethnicity*
replace ethnicity = "0" if ethnicity=="Not Hispanic/Latino"
replace ethnicity = "0" if ethnicity=="Non-Hispanic/Latino"
replace ethnicity = "0" if ethnicity=="Non-Hispanic"
```

```
replace ethnicity = "1" if ethnicity=="Hispanic/Latino"
destring ethnicity, replace
label define ethnicity_lab 0 "Non-Hispanic/Latino" 1 "Hispanic/Latino"
label values ethnicity ethnicity_lab
label variable ethnicity "Ethnicity"

*Race*
replace race = "0" if race=="White"
replace race = "1" if race=="Black or African American"
replace race = "2" if race=="Asian"
replace race = "3" if race=="American Indian/Alaska Native"
replace race = "3" if race=="American Indian or Alaska Native"
replace race = "4" if race=="Native Hawaiian/Pacific Islander"
replace race = "4" if race=="Native Hawaiian or Pacific Islander"
replace race = "4" if race=="Native Hawaiian or Other Pacific Islander"
replace race = "5" if race=="Other"
replace race = "6" if race=="Two or More Races"
destring race, replace
label define race_lab 0 "White" 1 "Black or African American" 2 "Asian" 3 "American Indian or Alaska Native" 4 "Native Hawaiian or Other Pacific Islander" 5 "Other" 6 "Two or More Races"
label values race race_lab
label variable race "Race"

*Household Annual Income*
replace income = "0" if income=="Less than $10,000"
replace income = "1" if income=="$10,000 to $19,999"
replace income = "2" if income=="$20,000 to $29,999"
replace income = "3" if income=="$30,000 to $39,999"
replace income = "4" if income=="$40,000 to $49,999"
replace income = "5" if income=="$50,000 to $59,999"
replace income = "6" if income=="$60,000 to $99,999"
replace income = "7" if income=="$100,000 to $149,999"
replace income = "8" if income=="$150,000 or more"
destring income, replace
label define income_lab 0 "Less than $10,000" 1 "$10,000 to $19,999" 2 "$20,000 to $29,999" 3 "$30,000 to $39,999" 4 "$40,000 to $49,999" 5 "$50,000 to $59,999" 6 "$60,000 to $99,999" 7 "$100,000 to $149,999" 8 "$150,000 or more"
label values income income_lab
label variable income "Total Household Annual Income (before taxes)"

*Homeownership - 2021*
replace homeown= "0" if homeown=="Rent"
replace homeown = "1" if homeown=="Own"
destring homeown, replace
label define homeown_lab 0 "Rent" 1 "Own"
label values homeown homeown_lab
label variable homeown "Homeownership"

count

** Check for multicolinearity among independent variables**
correlate atleast3_prep_action_gen awareness_gen awareness_pan genexpa c2_1g l1a race age homeown educ gender income census_region

*Table 1 - Weighted*
svyset _n, weight(weight) vce(linearized) singleunit(missing)

dtable i.atleast3_prep_action_gen i.awareness_gen i.awareness_pan i.genexpa i.c2_1g i.l1a i.race i.age i.homeown i.educ i.gender i.income i.census_region, by(atleast3_prep_action_pan) svy export(table1_weighted_bypandemicpreparednessstatus.docx, replace)

*Descriptive Statistics - weighted*
```

```
*Mean number of disaster preparedness actions*
svy: mean num_prep_action_general
svy: tabulate num_prep_action_general

foreach var of varlist prepb_a prepb_b prepb_c prepb_d prepb_e prepb_f prepb_g prepb_h prepb_i prepb_j prepb_k prepb_l prepb_m prepb_n prepb_o{
    svy: mean `var'
}

foreach var of varlist prepb_a prepb_b prepb_c prepb_d prepb_e prepb_f prepb_g prepb_h prepb_i prepb_j prepb_k prepb_l prepb_m prepb_n prepb_o{
    svy: tabulate `var'
}

*Mean number of pandemic preparedness actions*
svy: mean num_prep_action_pandemic
svy: tabulate num_prep_action_pandemic

foreach var of varlist prepa_a prepa_b prepa_c prepa_d prepa_e prepa_f prepa_g prepa_h prepa_i prepa_j prepa_k prepa_l prepa_m prepa_n prepa_o{
    svy: mean `var'
}

foreach var of varlist prepa_a prepa_b prepa_c prepa_d prepa_e prepa_f prepa_g prepa_h prepa_i prepa_j prepa_k prepa_l prepa_m prepa_n prepa_o{
    svy: tabulate `var'
}

*Number of people with at least 3 disaster preparedness actions*
svy: mean atleast3_prep_action_gen
svy: tabulate atleast3_prep_action_gen

*Number of people with at least 3 pandemic preparedness actions*
svy: mean atleast3_prep_action_pan
svy: tabulate atleast3_prep_action_pan

*Mean pandemic experience*
svy: mean genexpa
svy: tabulate genexpa

*Mean awareness of disaster information*
svy: mean awareness_gen
svy: tabulate awareness_gen

foreach var of varlist a3_a a3_b a3_c a3_d a3_e a3_f a3_g a3_h a3_i a3_j a3_k a3_l a3_m a3_n a3_o {
    svy: mean `var'
}

foreach var of varlist a3_a a3_b a3_c a3_d a3_e a3_f a3_g a3_h a3_i a3_j a3_k a3_l a3_m a3_n a3_o {
    svy: tabulate `var'
}

*Mean awareness of pandemic information*
svy: mean awareness_pan
svy: tabulate awareness_pan

foreach var of varlist a1_a a1_b a1_c a1_d a1_e a1_f a1_g a1_h a1_i a1_j a1_k{
    svy: mean `var'
}

foreach var of varlist a1_a a1_b a1_c a1_d a1_e a1_f a1_g a1_h a1_i a1_j a1_k{
    svy: tabulate `var'
}
```

```
*Table 2 - Weighted Logistic Regression*

*At Least 3 Preapredness Actions within the Past Year with Indicator Variables Weighted - Pandemic Preparedness (Yes, No)*
quietly svy: logistic  atleast3_prep_action_pan atleast3_prep_action_gen, or
estimates store model1
quietly svy: logistic  atleast3_prep_action_pan awareness_gen, or
estimates store model2
quietly svy: logistic  atleast3_prep_action_pan awareness_pan, or
estimates store model3
quietly svy: logistic  atleast3_prep_action_pan genexpa, or
estimates store model4
quietly svy: logistic  atleast3_prep_action_pan i.c2_1g, or
estimates store model5
quietly svy: logistic  atleast3_prep_action_pan i.l1a, or
estimates store model6
quietly svy: logistic atleast3_prep_action_pan i.race, or
estimates store model7
quietly svy: logistic  atleast3_prep_action_pan i.age, or
estimates store model8
quietly svy: logistic atleast3_prep_action_pan homeown, or
estimates store model9
quietly svy: logistic  atleast3_prep_action_pan i.educ, or
estimates store model10
quietly svy: logistic atleast3_prep_action_pan i.gender, or
estimates store model11
quietly svy: logistic  atleast3_prep_action_pan i.income, or
estimates store model12
quietly svy: logistic  atleast3_prep_action_pan i.census_region, or
estimates store model13

**Multivariate Pandemic Preparedness Weighted - All Variables - No Interaction**
quietly svy: logistic atleast3_prep_action_pan atleast3_prep_action_gen awareness_gen awareness_pan genexpa i.c2_1g i.l1a i.race i.age homeown i.educ i.gender i.income i.census_region, or
est store A

etable, estimates(model1 model2 model3 model4 model5 model6 model7 model8 model9 model10 model11 model12 model13) showstars showstarsnote stars(0.001 "***" 0.01 "**" 0.05 "*") title("Table 2a: Univariate models for FEMA 2021") export(fema2021_nhs_unireg.docx, replace)

*Investigating Interaction Term - Disaster Preparedness and Census Region*
gen dis_prep_census_region=atleast3_prep_action_gen*census_region
label variable dis_prep_census_region "Interaction between Disaster Preparedness and Census Region"

** Multivariate Pandemic Preparedness Weighted - All Variables with Interaction**
quietly svy: logistic  atleast3_prep_action_pan atleast3_prep_action_gen awareness_gen awareness_pan genexpa i.c2_1g i.l1a i.race i.age homeown i.educ i.gender i.income i.census_region dis_prep_census_region, or
est store B

** Only include covariates with at least one significant category from the univariate analysis**

* Homeownership was removed because none of the subcategories of these variables had a significant result for the univariate regression. *

**Multivariate Pandemic Preparedness Weighted Significant Covariates with Interaction*
quietly svy: logistic atleast3_prep_action_pan atleast3_prep_action_gen awareness_gen awareness_pan genexpa i.c2_1g i.l1a i.race i.age i.educ i.gender i.income i.census_region dis_prep_census_region, or
est store C
estimates store model14
estat gof

```

```
* The interaction term was removed because it was not found to be significant across all study years *

**Multivariate Pandemic Preparedness Weighted Significant Covariates across all three years - removed homeownership, income, census region, and the interaction term**
quietly svy: logistic atleast3_prep_action_pan atleast3_prep_action_gen awareness_gen awareness_pan genexpa i.c2_1g i.l1a i.race i.age i.educ i.gender, or
estimates store model15
est store D
est stats *
estat gof

etable, estimates(model14 model15) showstars showstarsnote stars(0.001 "***" 0.01 "**" 0.05 "*") title("Table 2a: Multivariate models for FEMA 2021") export(fema2021_nhs_multireg.docx, replace)

save "C:\Location\fema_national_household_survey_data_and_codebook_results_2021.dta", replace

*-------------------------------------------------------------------
clear

***2022 NHS Dataset***

tempfile temp_data
import excel "C:\Location\fema_national_household_survey_2022_data_and_codebook.xlsx", sheet("2022 NHS General Data") firstrow clear
export delimited using `temp_data', replace
import delimited `temp_data', varnames(2) clear

save "C:\Location\fema_national_household_survey_2022_data_and_codebook.dta", replace

*Number of Observations Prior to Data Cleaning*
count

*Disaster Data*

*Awareness of Disaster Preparedness Information - 2022*

foreach var of varlist a3_a a3_b a3_c a3_d a3_e a3_f a3_g a3_h a3_i a3_j a3_k a3_l {
    replace `var' = "1" if `var' != "Blank"
}

foreach var of varlist a3_a a3_b a3_c a3_d a3_e a3_f a3_g a3_h a3_i a3_j a3_k a3_l a3_m a3_n a3_o{
    replace `var' = "0" if `var' == "Blank"
}
replace a3_m = "0" if a3_m=="None"
replace a3_m = "0" if a3_m=="None of the above"
drop if strpos(a3_n, "Don't know")
drop if strpos(a3_o, "Prefer not to answer")
destring a3_a a3_b a3_c a3_d a3_e a3_f a3_g a3_h a3_i a3_j a3_k a3_l a3_m a3_n a3_o, replace
egen awareness_gen = rowtotal(a3_a a3_b a3_c a3_d a3_e a3_f a3_g a3_h a3_i a3_j a3_k a3_l)
tabulate awareness_gen
replace awareness_gen = 1 if awareness_gen>= 1
replace awareness_gen = 0 if awareness_gen< 1
label define awareness_gen_lab 0 "No" 1 "Yes"
label values awareness_gen awareness_gen_lab
label variable awareness_gen "Awareness of Disaster Preparedness Information"
tabulate awareness_gen

*Number of Disaster Preparedness Actions within the Last Year and At Least Three Disaster Preparedness Actions - 2022*
foreach var of varlist prepb_a prepb_b prepb_c prepb_d prepb_e prepb_f prepb_g prepb_h prepb_i prepb_j prepb_k prepb_l {
    replace `var' = "1" if `var' != "Blank"
}
```

```

foreach var of varlist prepb_a prepb_b prepb_c prepb_d prepb_e prepb_f prepb_g prepb_h prepb_i prepb_j prepb_k prepb_l prepb_m prepb_n prepb_o{
    replace `var' = "0" if `var' == "Blank"
}
replace prepb_m = "0" if prepb_m=="None"
replace prepb_m = "0" if prepb_m=="None of the above"
drop if strpos(prepb_n, "Don't know")
drop if strpos(prepb_o, "Prefer not to answer")
destring prepb_a prepb_b prepb_c prepb_d prepb_e prepb_f prepb_g prepb_h prepb_i prepb_j prepb_k prepb_l prepb_m prepb_n prepb_o, replace
egen num_prep_action_general = rowtotal(prepb_a prepb_b prepb_c prepb_d prepb_e prepb_f prepb_g prepb_h prepb_i prepb_j prepb_k prepb_l)
label variable num_prep_action_general "Number of Disaster Preparedness Actions within the Last Year"
summarize num_prep_action_general
tabulate num_prep_action_general
gen atleast3_prep_action_gen = num_prep_action_general
replace atleast3_prep_action_gen = 0 if atleast3_prep_action_gen== 0 | atleast3_prep_action_gen== 1 | atleast3_prep_action_gen== 2
replace atleast3_prep_action_gen = 1 if inrange(atleast3_prep_action_gen, 3, 12)
label define atleast3_prep_action_gen_lab 0 "Unprepared" 1 "Prepared"
label values atleast3_prep_action_gen atleast3_prep_action_gen_lab
label variable atleast3_prep_action_gen "At Least 3 Disaster Preparedness Actions in the Past Year"
tabulate atleast3_prep_action_gen

*Outcome - Pandemic Preparedness Actions within the Last Year*
*Number of Pandemic Preparedness Actions within the Last Year and At Least Three Pandemic Preparedness Actions - 2022*
foreach var of varlist prepa_a prepa_b prepa_c prepa_d prepa_e prepa_f prepa_g prepa_h prepa_i prepa_j prepa_k prepa_l {
    replace `var' = "1" if `var' != "Blank"
}

foreach var of varlist prepa_a prepa_b prepa_c prepa_d prepa_e prepa_f prepa_g prepa_h prepa_i prepa_j prepa_k prepa_l prepa_m prepa_n prepa_o{
    replace `var' = "0" if `var' == "Blank"
}
replace prepa_m = "0" if prepa_m=="None"
replace prepa_m = "0" if prepa_m=="None of the above"
drop if strpos(prepa_n, "Don't know")
drop if strpos(prepa_o, "Prefer not to answer")
destring prepa_a prepa_b prepa_c prepa_d prepa_e prepa_f prepa_g prepa_h prepa_i prepa_j prepa_k prepa_l prepa_m prepa_n prepa_o, replace
egen num_prep_action_pandemic = rowtotal(prepa_a prepa_b prepa_c prepa_d prepa_e prepa_f prepa_g prepa_h prepa_i prepa_j prepa_k prepa_l)
label variable num_prep_action_pandemic "Number of Pandemic Preparedness Actions within the Last Year"
summarize num_prep_action_pandemic
tabulate num_prep_action_pandemic
gen atleast3_prep_action_pan = num_prep_action_pandemic
replace atleast3_prep_action_pan = 0 if atleast3_prep_action_pan== 0 | atleast3_prep_action_pan== 1 | atleast3_prep_action_pan== 2
replace atleast3_prep_action_pan = 1 if inrange(atleast3_prep_action_pan, 3, 12)
label define atleast3_prep_action_pan_lab 0 "Unprepared" 1 "Prepared"
label values atleast3_prep_action_pan atleast3_prep_action_pan_lab
label variable atleast3_prep_action_pan "At Least 3 Pandemic Preparedness Actions in the Past Year"
tabulate atleast3_prep_action_pan

*FEMA's Four Influencers of Preparedness - Pandemics*

*Pandemic Risk Perception*
drop if strpos(l1a, "Don't know")
drop if strpos(l1a, "Prefer not to answer")
replace l1a = "0" if l1a=="Unlikely"
```

```
replace l1a = "1" if l1a=="Likely"
replace l1a = "2" if l1a=="Very likely"
replace l1a = "2" if l1a=="Very Likely"
destring l1a, replace
label define l1a_lab 0 "Unlikely" 1 "Likely" 2 "Very likely"
label values l1a l1a_lab
label variable l1a "Pandemic Risk Perception"

*Previous Pandemic Experience*
drop if strpos(genexpa, "Don't know")
drop if strpos(genexpa, "Prefer not to answer")
replace genexpa ="0" if genexpa=="No"
replace genexpa ="1" if genexpa=="Yes"
replace genexpa ="1" if genexpa=="Yes, COVID-19"
replace genexpa ="1" if genexpa=="Yes, COVID-19 and something else"
destring genexpa, replace
label define genexpa_lab 0 "No" 1 "Yes"
label values genexpa genexpa_lab
label variable genexpa "Previous Pandemic Experience"

*Pandemic Confidence/Self-efficacy*
drop if strpos(c2_1g, "Don't know")
drop if strpos(c2_1g, "Prefer not to answer")
replace c2_1g = "1" if c2_1g=="Not at all confident"
replace c2_1g = "2" if c2_1g=="Slightly confident"
replace c2_1g = "3" if c2_1g=="Somewhat confident"
replace c2_1g = "4" if c2_1g=="Moderately confident"
replace c2_1g = "5" if c2_1g=="Extremely confident"
destring c2_1g, replace
label define c2_1g_lab 1 "Not at all confident" 2 "Slightly confident" 3 "Somewhat confident" 4 "Moderately confident" 5 "Extremely confident"
label values c2_1g c2_1g_lab
label variable c2_1g "Pandemic Confidence/Self-efficacy"

*Awareness of Pandemic Preparedness Information - 2022*

foreach var of varlist a1_a a1_b a1_c a1_d a1_e a1_f a1_g a1_h{
    replace `var' = "1" if `var' != "Blank"
}

foreach var of varlist a1_a a1_b a1_c a1_d a1_e a1_f a1_g a1_h a1_i a1_j a1_k{
    replace `var' = "0" if `var' == "Blank"
}
replace a1_i = "0" if a1_i=="None"
replace a1_i = "0" if a1_i=="None of the above"
drop if strpos(a1_j, "Don't know")
drop if strpos(a1_k, "Prefer not to answer")
destring a1_a a1_b a1_c a1_d a1_e a1_f a1_g a1_h a1_i a1_j a1_k, replace
egen awareness_pan = rowtotal(a1_a a1_b a1_c a1_d a1_e a1_f a1_g a1_h)
tabulate awareness_pan
replace awareness_pan = 1 if awareness_pan>= 1
replace awareness_pan = 0 if awareness_pan< 1
label define awareness_pan_lab 0 "No" 1 "Yes"
label values awareness_pan awareness_pan_lab
label variable awareness_pan "Awareness of Pandemic Preparedness Information"
tabulate awareness_pan

*Demographic Information*

*Census Region*
replace census_region ="1" if census_region=="West"
replace census_region ="2" if census_region=="Midwest"
replace census_region ="3" if census_region=="Northeast"
```

```
replace census_region ="4" if census_region=="South"
replace census_region ="5" if census_region==""
replace census_region ="5" if census_region=="N/A"
replace census_region ="5" if census_region=="Territories"
destring census_region, replace
label define census_region_lab 1 "West" 2 "Midwest" 3 "Northeast" 4 "South" 5 "Territories"
label values census_region census_region_lab
label variable census_region "Census Region"

*Age* 2022
replace age = "0" if age=="18-19"
replace age = "0" if age=="20-29"
replace age = "1" if age=="30-39"
replace age = "2" if age=="40-49"
replace age = "3" if age=="50-59"
replace age = "4" if age=="60-69"
replace age = "5" if age=="70-79"
replace age = "6" if age=="Over 80"
replace age = "6" if age=="80+"
destring age, replace
label define age_lab 0 "18-29" 1 "30-39" 2 "40-49" 3 "50-59" 4 "60-69" 5 "70-79" 6 "Over 80"
label values age age_lab
label variable age "Age"

*Gender*
replace sex = "0" if sex=="Male"
replace sex = "1" if sex=="Female"
replace sex = "2" if sex=="Third-Gender/Other"
replace sex = "2" if sex=="Third-gender/Other"
destring sex, replace
label define sex_lab 0 "Male" 1 "Female" 2 "Third Gender/Other"
label values sex sex_lab
label variable sex "Gender"

*Education*
replace education = "0" if education=="Less than high school diploma"
replace education = "1" if education=="High school degree or diploma"
replace education = "2" if education=="Some college, no degree"
replace education = "3" if education=="Associate's degree"
replace education = "4" if education=="Bachelor's degree"
replace education = "5" if education=="Post graduate work/degree or professional degree"
destring education, replace
label define education_lab 0 "Less than high school diploma" 1 "High school degree or diploma" 2 "Some college, no degree" 3 "Associate's degree" 4 "Bachelor's degree" 5 "Post graduate work/degree or professional degree"
label values education education_lab
label variable education "Education"


*Ethnicity*
replace ethnicity = "0" if ethnicity=="Not Hispanic/Latino"
replace ethnicity = "0" if ethnicity=="Non-Hispanic/Latino"
replace ethnicity = "0" if ethnicity=="Non-Hispanic"
replace ethnicity = "1" if ethnicity=="Hispanic/Latino"
destring ethnicity, replace
label define ethnicity_lab 0 "Non-Hispanic/Latino" 1 "Hispanic/Latino"
label values ethnicity ethnicity_lab
label variable ethnicity "Ethnicity"

*Race*
replace race = "0" if race=="White"
replace race = "1" if race=="Black or African American"
replace race = "2" if race=="Asian"
```

```
replace race = "3" if race=="American Indian/Alaska Native"
replace race = "3" if race=="American Indian or Alaska Native"
replace race = "4" if race=="Native Hawaiian/Pacific Islander"
replace race = "4" if race=="Native Hawaiian or Pacific Islander"
replace race = "4" if race=="Native Hawaiian or Other Pacific Islander"
replace race = "5" if race=="Other"
replace race = "6" if race=="Two or More Races"
destring race, replace
label define race_lab 0 "White" 1 "Black or African American" 2 "Asian" 3 "American Indian or Alaska 
Native" 4 "Native Hawaiian or Other Pacific Islander" 5 "Other" 6 "Two or More Races"
label values race race_lab
label variable race "Race"

*Household Annual Income*
replace income = "0" if income=="Less than $10,000"
replace income = "1" if income=="$10,000 to $19,999"
replace income = "2" if income=="$20,000 to $29,999"
replace income = "3" if income=="$30,000 to $39,999"
replace income = "4" if income=="$40,000 to $49,999"
replace income = "5" if income=="$50,000 to $59,999"
replace income = "6" if income=="$60,000 to $99,999"
replace income = "7" if income=="$100,000 to $149,999"
replace income = "8" if income=="$150,000 or more"
destring income, replace
label define income_lab 0 "Less than $10,000" 1 "$10,000 to $19,999" 2 "$20,000 to $29,999" 3 
"$30,000 to $39,999" 4 "$40,000 to $49,999" 5 "$50,000 to $59,999" 6 "$60,000 to $99,999" 7 
"$100,000 to $149,999" 8 "$150,000 or more"
label values income income_lab
label variable income "Total Household Annual Income (before taxes)"

*Homeownership*
replace homeownership = "0" if homeownership=="Rent"
replace homeownership = "1" if homeownership=="Own"
destring homeownership, replace
label define homeownership_lab 0 "Rent" 1 "Own"
label values homeownership homeownership_lab
label variable homeownership "Homeownership"

*Rurality - only available for 2022 and 2023*
replace rurality = "0" if rurality=="Rural"
replace rurality = "1" if rurality=="Urban"
replace rurality = "1" if rurality=="Urban Cluster"
destring rurality, replace
label define rurality_lab 0 "Rural" 1 "Urban"
label values rurality rurality_lab
label variable rurality "Rurality Calculated using ZIP code, County, and State"

count

** Check for multicolinearity among independent variables**
correlate atleast3_prep_action_gen awareness_gen awareness_pan genexpa c2_1g l1a race age 
homeownership education sex income census_region

*Table 1 - Weighted*
svyset _n, weight(weight) vce(linearized) singleunit(missing)

dtable i.atleast3_prep_action_gen i.awareness_gen i.awareness_pan i.genexpa i.c2_1g i.l1a i.race i.
age i.homeownership i.education i.sex i.income i.census_region, by(atleast3_prep_action_pan) svy 
export(NHS2022_table1_weighted_bypandemicpreparednessstatus.docx, replace)

*Descriptive Statistics - weighted*

*Mean number of disaster preparedness actions*
```

```
svy: mean num_prep_action_general
svy: tabulate num_prep_action_general

foreach var of varlist prepb_a prepb_b prepb_c prepb_d prepb_e prepb_f prepb_g prepb_h prepb_i prepb_j prepb_k prepb_l prepb_m prepb_n prepb_o{
    svy: mean `var'
}

foreach var of varlist prepb_a prepb_b prepb_c prepb_d prepb_e prepb_f prepb_g prepb_h prepb_i prepb_j prepb_k prepb_l prepb_m prepb_n prepb_o{
    svy: tabulate `var'
}

*Mean number of pandemic preparedness actions*
svy: mean num_prep_action_pandemic
svy: tabulate num_prep_action_pandemic

foreach var of varlist prepa_a prepa_b prepa_c prepa_d prepa_e prepa_f prepa_g prepa_h prepa_i prepa_j prepa_k prepa_l prepa_m prepa_n prepa_o{
    svy: mean `var'
}

foreach var of varlist prepa_a prepa_b prepa_c prepa_d prepa_e prepa_f prepa_g prepa_h prepa_i prepa_j prepa_k prepa_l prepa_m prepa_n prepa_o{
    svy: tabulate `var'
}

*Number of people with at least 3 disaster preparedness actions*
svy: mean atleast3_prep_action_gen
svy: tabulate atleast3_prep_action_gen

*Number of people with at least 3 pandemic preparedness actions*
svy: mean atleast3_prep_action_pan
svy: tabulate atleast3_prep_action_pan

*Mean pandemic experience*
svy: mean genexpa
svy: tabulate genexpa

*Mean awareness of disaster information*
svy: mean awareness_gen
svy: tabulate awareness_gen

foreach var of varlist a3_a a3_b a3_c a3_d a3_e a3_f a3_g a3_h a3_i a3_j a3_k a3_l a3_m a3_n a3_o {
    svy: mean `var'
}

foreach var of varlist a3_a a3_b a3_c a3_d a3_e a3_f a3_g a3_h a3_i a3_j a3_k a3_l a3_m a3_n a3_o{
svy: tabulate `var'
}

*Mean awareness of pandemic information*
svy: mean awareness_pan
svy: tabulate awareness_pan

foreach var of varlist a1_a a1_b a1_c a1_d a1_e a1_f a1_g a1_h a1_i a1_j a1_k{
    svy: mean `var'
}

foreach var of varlist a1_a a1_b a1_c a1_d a1_e a1_f a1_g a1_h a1_i a1_j a1_k{
    svy: tabulate `var'
}
```

```
*Table 2 - Weighted Logistic Regression*

*At Least 3 Preapredness Actions within the Past Year with Indicator Variables Weighted - Pandemic Preparedness (Yes, No)*
quietly svy: logistic  atleast3_prep_action_pan atleast3_prep_action_gen, or
estimates store model1
quietly svy: logistic  atleast3_prep_action_pan awareness_gen, or
estimates store model2
quietly svy: logistic  atleast3_prep_action_pan awareness_pan, or
estimates store model3
quietly svy: logistic  atleast3_prep_action_pan genexpa, or
estimates store model4
quietly svy: logistic  atleast3_prep_action_pan i.c2_1g, or
estimates store model5
quietly svy: logistic  atleast3_prep_action_pan i.l1a, or
estimates store model6
quietly svy: logistic atleast3_prep_action_pan i.race, or
estimates store model7
quietly svy: logistic  atleast3_prep_action_pan i.age, or
estimates store model8
quietly svy: logistic atleast3_prep_action_pan homeownership, or
estimates store model9
quietly svy: logistic  atleast3_prep_action_pan i.education, or
estimates store model10
quietly svy: logistic atleast3_prep_action_pan i.sex, or
estimates store model11
quietly svy: logistic  atleast3_prep_action_pan i.income, or
estimates store model12
quietly svy: logistic  atleast3_prep_action_pan i.census_region, or
estimates store model13

**Multivariate Pandemic Preparedness Weighted - All Variables - No Interaction**
quietly svy: logistic atleast3_prep_action_pan atleast3_prep_action_gen awareness_gen awareness_pan genexpa i.c2_1g i.l1a i.race i.age homeownership i.education i.sex i.income i.census_region, or
est store A

etable, estimates(model1 model2 model3 model4 model5 model6 model7 model8 model9 model10 model11 model12 model13) showstars showstarsnote stars(0.001 "***" 0.01 "**" 0.05 "*") title("Table 2a: Univariate models for FEMA 2022") export(fema2022_nhs_unireg.docx, replace)

*Investigating Interaction Term - Disaster Preparedness and Census Region*
gen dis_prep_census_region=atleast3_prep_action_gen*census_region
label variable dis_prep_census_region "Interaction between Disaster Preparedness and Census Region"

** Multivariate Pandemic Preparedness Weighted - All Variables with Interaction **
quietly svy: logistic  atleast3_prep_action_pan atleast3_prep_action_gen awareness_gen awareness_pan genexpa i.c2_1g i.l1a i.race i.age homeownership i.education i.sex i.income i.census_region dis_prep_census_region, or
est store B

** Only include covariates with at least one significant category from the univariate analysis**

* Homeownership and income were removed because none of the subcategories of these variables had a significant result for the univariate regression. Although census region was not significant for any of its subcategories in the univariate analysis, it was included in order to explore the interaction between census region *

**Multivariate Pandemic Preparedness Weighted Significant Covariates with Interaction*
quietly svy: logistic atleast3_prep_action_pan atleast3_prep_action_gen awareness_gen awareness_pan genexpa i.c2_1g i.l1a i.race i.age i.education i.sex i.census_region dis_prep_census_region, or
est store C
estimates store model14
estat gof
```

```

* The interaction term was removed because it was not found to be significant across all study years *

**Multivariate Pandemic Preparedness Weighted Significant Covariates across all three years - removed homeownership, income, census region, and the interaction term**
svy: logistic atleast3_prep_action_pan atleast3_prep_action_gen awareness_gen awareness_pan genexpa i.c2_1g i.l1a i.race i.age i.education i.sex, or
estimates store model15
est store D
est stats *
estat gof

etable, estimates(model14 model15) showstars showstarsnote stars(0.001 "***" 0.01 "**" 0.05 "*") title("Table 2a: Multivariate models for FEMA 2022") export(fema2022_nhs_multireg.docx, replace)

save "C:\Location\fema_national_household_survey_2022_data_and_codebook_results.dta", replace

clear
*--------------------------------------------------------------------

***2023 NHS Dataset***

tempfile temp_data
import excel "C:\Location\fema_national_household_survey_2023_data_and_codebook.xlsx", sheet("Core Survey") firstrow clear
export delimited using `temp_data', replace
import delimited `temp_data', varnames(2) clear

save "C:\Location\fema_national_household_survey_2023_data_and_codebook.dta", replace

*Number of Observations Prior to Data Cleaning*
count

*Disaster Data*

*Awareness of Disaster Preparedness Information - 2023
foreach var of varlist dis_awareness_a dis_awareness_b dis_awareness_c dis_awareness_d dis_awareness_e dis_awareness_f dis_awareness_g dis_awareness_h dis_awareness_i dis_awareness_j dis_awareness_k dis_awareness_l {
    replace `var' = "1" if `var' != "Blank"
}

foreach var of varlist dis_awareness_a dis_awareness_b dis_awareness_c dis_awareness_d dis_awareness_e dis_awareness_f dis_awareness_g dis_awareness_h dis_awareness_i dis_awareness_j dis_awareness_k dis_awareness_l dis_awareness_m dis_awareness_n{
    replace `var' = "0" if `var' == "Blank"
}
replace dis_awareness_m = "0" if dis_awareness_m=="None"
replace dis_awareness_m = "0" if dis_awareness_m=="None of the above"
drop if strpos(dis_awareness_n, "Don't know")
destring dis_awareness_a dis_awareness_b dis_awareness_c dis_awareness_d dis_awareness_e dis_awareness_f dis_awareness_g dis_awareness_h dis_awareness_i dis_awareness_j dis_awareness_k dis_awareness_l dis_awareness_m dis_awareness_n, replace
foreach var of varlist dis_awareness_a dis_awareness_b dis_awareness_c dis_awareness_d dis_awareness_e dis_awareness_f dis_awareness_g dis_awareness_h dis_awareness_i dis_awareness_j dis_awareness_k dis_awareness_l {
   sum `var'
}
foreach var of varlist dis_awareness_a dis_awareness_b dis_awareness_c dis_awareness_d dis_awareness_e dis_awareness_f dis_awareness_g dis_awareness_h dis_awareness_i dis_awareness_j dis_awareness_k dis_awareness_l {
   tabulate `var'
}
```

```
egen awareness_gen = rowtotal(dis_awareness_a dis_awareness_b dis_awareness_c dis_awareness_d dis_awareness_e dis_awareness_f dis_awareness_g dis_awareness_h dis_awareness_i dis_awareness_j dis_awareness_k dis_awareness_l)
tabulate awareness_gen
replace awareness_gen = 1 if awareness_gen>= 1
replace awareness_gen = 0 if awareness_gen< 1
label define awareness_gen_lab 0 "No" 1 "Yes"
label values awareness_gen awareness_gen_lab
label variable awareness_gen "Awareness of Disaster Preparedness Information"
tabulate awareness_gen

*Number of Disaster Preparedness Actions within the Last Year and At Least Three Disaster Preparedness Actions - 2023*
foreach var of varlist dis_prepactions_a dis_prepactions_b dis_prepactions_c dis_prepactions_d dis_prepactions_e dis_prepactions_f dis_prepactions_g dis_prepactions_h dis_prepactions_i dis_prepactions_j dis_prepactions_k dis_prepactions_l {
    replace `var' = "1" if `var' != "Blank"
}

foreach var of varlist dis_prepactions_a dis_prepactions_b dis_prepactions_c dis_prepactions_d dis_prepactions_e dis_prepactions_f dis_prepactions_g dis_prepactions_h dis_prepactions_i dis_prepactions_j dis_prepactions_k dis_prepactions_l dis_prepactions_m dis_prepactions_n{
    replace `var' = "0" if `var' == "Blank"
}
replace dis_prepactions_m = "0" if dis_prepactions_m=="None"
replace dis_prepactions_m = "0" if dis_prepactions_m=="None of the above"
drop if strpos(dis_prepactions_n, "Don't know")
destring dis_prepactions_a dis_prepactions_b dis_prepactions_c dis_prepactions_d dis_prepactions_e dis_prepactions_f dis_prepactions_g dis_prepactions_h dis_prepactions_i dis_prepactions_j dis_prepactions_k dis_prepactions_l dis_prepactions_m dis_prepactions_n, replace
egen num_prep_action_general = rowtotal(dis_prepactions_a dis_prepactions_b dis_prepactions_c dis_prepactions_d dis_prepactions_e dis_prepactions_f dis_prepactions_g dis_prepactions_h dis_prepactions_i dis_prepactions_j dis_prepactions_k dis_prepactions_l)
label variable num_prep_action_general "Number of Disaster Preparedness Actions within the Last Year"
summarize num_prep_action_general
tabulate num_prep_action_general
gen atleast3_prep_action_gen = num_prep_action_general
replace atleast3_prep_action_gen = 0 if atleast3_prep_action_gen== 0 | atleast3_prep_action_gen== 1 | atleast3_prep_action_gen== 2
replace atleast3_prep_action_gen = 1 if inrange(atleast3_prep_action_gen, 3, 12)
label define atleast3_prep_action_gen_lab 0 "Unprepared" 1 "Prepared"
label values atleast3_prep_action_gen atleast3_prep_action_gen_lab
label variable atleast3_prep_action_gen "At Least 3 Disaster Preparedness Actions in the Past Year"
tabulate atleast3_prep_action_gen

*Outcome - Pandemic Preparedness Actions within the Last Year*

*Number of Pandemic Preparedness Actions within the Last Year and At Least Three Pandemic Preparedness Actions - 2023*
foreach var of varlist pan_prepactions_a pan_prepactions_b pan_prepactions_c pan_prepactions_d pan_prepactions_e pan_prepactions_f pan_prepactions_g pan_prepactions_h pan_prepactions_i pan_prepactions_j pan_prepactions_k pan_prepactions_l {
    replace `var' = "1" if `var' != "Blank"
}

foreach var of varlist pan_prepactions_a pan_prepactions_b pan_prepactions_c pan_prepactions_d pan_prepactions_e pan_prepactions_f pan_prepactions_g pan_prepactions_h pan_prepactions_i pan_prepactions_j pan_prepactions_k pan_prepactions_l pan_prepactions_m pan_prepactions_n{
    replace `var' = "0" if `var' == "Blank"
}
replace pan_prepactions_m = "0" if pan_prepactions_m=="None"
replace pan_prepactions_m = "0" if pan_prepactions_m=="None of the above"
drop if strpos(pan_prepactions_n, "Don't know")
```

```
destring pan_prepactions_a pan_prepactions_b pan_prepactions_c pan_prepactions_d pan_prepactions_e pan_prepactions_f pan_prepactions_g pan_prepactions_h pan_prepactions_i pan_prepactions_j pan_prepactions_k pan_prepactions_l pan_prepactions_m pan_prepactions_n, replace
egen num_prep_action_pandemic = rowtotal(pan_prepactions_a pan_prepactions_b pan_prepactions_c pan_prepactions_d pan_prepactions_e pan_prepactions_f pan_prepactions_g pan_prepactions_h pan_prepactions_i pan_prepactions_j pan_prepactions_k pan_prepactions_l)
label variable num_prep_action_pandemic "Number of Pandemic Preparedness Actions within the Last Year"
tabulate num_prep_action_pandemic
gen atleast3_prep_action_pan = num_prep_action_pandemic
replace atleast3_prep_action_pan = 0 if atleast3_prep_action_pan== 0 | atleast3_prep_action_pan== 1 | atleast3_prep_action_pan== 2
replace atleast3_prep_action_pan = 1 if inrange(atleast3_prep_action_pan, 3, 12)
label define atleast3_prep_action_pan_lab 0 "Unprepared" 1 "Prepared"
label values atleast3_prep_action_pan atleast3_prep_action_pan_lab
label variable atleast3_prep_action_pan "At Least 3 Pandemic Preparedness Actions in the Past Year"
tabulate atleast3_prep_action_pan

*FEMA's Four Influencers of Preparedness - Pandemics*

*Pandemic Risk Perception*
drop if strpos(pan_perception, "Don't know")
drop if strpos(pan_perception, "Prefer not to answer")
replace pan_perception = "0" if pan_perception=="Unlikely"
replace pan_perception = "1" if pan_perception=="Likely"
replace pan_perception = "2" if pan_perception=="Very likely"
replace pan_perception = "2" if pan_perception=="Very Likely"
destring pan_perception, replace
label define pan_perception_lab 0 "Unlikely" 1 "Likely" 2 "Very likely"
label values pan_perception pan_perception_lab
label variable pan_perception "Pandemic Risk Perception"

*Previous Pandemic Experience*
drop if strpos(pan_exp, "Don't know")
drop if strpos(pan_exp, "Prefer not to answer")
replace pan_exp ="0" if pan_exp=="No"
replace pan_exp ="1" if pan_exp=="Yes"
replace pan_exp ="1" if pan_exp=="Yes, COVID-19"
replace pan_exp ="1" if pan_exp=="Yes, COVID-19 and something else"
destring pan_exp, replace
label define pan_exp_lab 0 "No" 1 "Yes"
label values pan_exp pan_exp_lab
label variable pan_exp "Previous Pandemic Experience"

*Pandemic Confidence/Self-efficacy*
drop if strpos(pan_confidence, "Don't know")
drop if strpos(pan_confidence, "Prefer not to answer")
replace pan_confidence = "1" if pan_confidence=="Not at all confident"
replace pan_confidence = "2" if pan_confidence=="Slightly confident"
replace pan_confidence = "3" if pan_confidence=="Somewhat confident"
replace pan_confidence = "4" if pan_confidence=="Moderately confident"
replace pan_confidence = "5" if pan_confidence=="Extremely confident"
destring pan_confidence, replace
label define pan_confidence_lab 1 "Not at all confident" 2 "Slightly confident" 3 "Somewhat confident" 4 "Moderately confident" 5 "Extremely confident"
label values pan_confidence pan_confidence_lab
label variable pan_confidence "Pandemic Confidence/Self-efficacy"

*Awareness of Pandemic Preparedness Information - 2023
foreach var of varlist pan_awareness_a pan_awareness_b pan_awareness_c pan_awareness_d pan_awareness_e pan_awareness_f pan_awareness_g pan_awareness_h{
    replace `var' = "1" if `var' != "Blank"
}

```

```
foreach var of varlist pan_awareness_a pan_awareness_b pan_awareness_c pan_awareness_d
pan_awareness_e pan_awareness_f pan_awareness_g pan_awareness_h pan_awareness_i pan_awareness_j{
    replace `var' = "0" if `var' == "Blank"
}
replace pan_awareness_i = "0" if pan_awareness_i=="None"
replace pan_awareness_i = "0" if pan_awareness_i=="None of the above"
drop if strpos(pan_awareness_j, "Don't know")
destring pan_awareness_a pan_awareness_b pan_awareness_c pan_awareness_d pan_awareness_e
pan_awareness_f pan_awareness_g pan_awareness_h pan_awareness_i pan_awareness_j, replace
foreach var of varlist pan_awareness_a pan_awareness_b pan_awareness_c pan_awareness_d
pan_awareness_e pan_awareness_f pan_awareness_g pan_awareness_h pan_awareness_i pan_awareness_j{
    summarize `var'
}
foreach var of varlist pan_awareness_a pan_awareness_b pan_awareness_c pan_awareness_d
pan_awareness_e pan_awareness_f pan_awareness_g pan_awareness_h pan_awareness_i pan_awareness_j{
   tabulate `var'
}
egen awareness_pan = rowtotal(pan_awareness_a pan_awareness_b pan_awareness_c pan_awareness_d
pan_awareness_e pan_awareness_f pan_awareness_g pan_awareness_h)
tabulate awareness_pan
replace awareness_pan = 1 if awareness_pan>= 1
replace awareness_pan = 0 if awareness_pan< 1
label define awareness_pan_lab 0 "No" 1 "Yes"
label values awareness_pan awareness_pan_lab
label variable awareness_pan "Awareness of Pandemic Preparedness Information"
tabulate awareness_pan

*Demographic Information*

*Census Region*
replace census_region ="1" if census_region=="West"
replace census_region ="2" if census_region=="Midwest"
replace census_region ="3" if census_region=="Northeast"
replace census_region ="4" if census_region=="South"
replace census_region ="5" if census_region==""
replace census_region ="5" if census_region=="N/A"
replace census_region ="5" if census_region=="Territories"
destring census_region, replace
label define census_region_lab 1 "West" 2 "Midwest" 3 "Northeast" 4 "South" 5 "Territories"
label values census_region census_region_lab
label variable census_region "Census Region"

*Age*
replace age = "0" if age=="18-19"
replace age = "0" if age=="20-29"
replace age = "1" if age=="30-39"
replace age = "2" if age=="40-49"
replace age = "3" if age=="50-59"
replace age = "4" if age=="60-69"
replace age = "5" if age=="70-79"
replace age = "6" if age=="Over 80"
replace age = "6" if age=="80+"
destring age, replace
label define age_lab 0 "18-29" 1 "30-39" 2 "40-49" 3 "50-59" 4 "60-69" 5 "70-79" 6 "Over 80"
label values age age_lab
label variable age "Age"

*Gender*
replace sex = "0" if sex=="Male"
replace sex = "1" if sex=="Female"
replace sex = "2" if sex=="Third-Gender/Other"
replace sex = "2" if sex=="Third-gender/Other"
destring sex, replace
```

```
label define sex_lab 0 "Male" 1 "Female" 2 "Third Gender/Other"
label values sex sex_lab
label variable sex "Gender"

*Education*
replace education = "0" if education=="Less than high school diploma"
replace education = "1" if education=="High school degree or diploma"
replace education = "2" if education=="Some college, no degree"
replace education = "3" if education=="Associate's degree"
replace education = "4" if education=="Bachelor's degree"
replace education = "5" if education=="Post graduate work/degree or professional degree"
destring education, replace
label define education_lab 0 "Less than high school diploma" 1 "High school degree or diploma" 2 "Some college, no degree" 3 "Associate's degree" 4 "Bachelor's degree" 5 "Post graduate work/degree or professional degree"
label values education education_lab
label variable education "Education"


*Ethnicity*
replace ethnicity = "0" if ethnicity=="Not Hispanic/Latino"
replace ethnicity = "0" if ethnicity=="Non-Hispanic/Latino"
replace ethnicity = "0" if ethnicity=="Non-Hispanic"
replace ethnicity = "1" if ethnicity=="Hispanic/Latino"
destring ethnicity, replace
label define ethnicity_lab 0 "Non-Hispanic/Latino" 1 "Hispanic/Latino"
label values ethnicity ethnicity_lab
label variable ethnicity "Ethnicity"

*Race - 2023*
replace race_selfid = "0" if race_selfid=="White"
replace race_selfid = "1" if race_selfid=="Black or African American"
replace race_selfid = "2" if race_selfid=="Asian"
replace race_selfid = "3" if race_selfid=="American Indian/Alaska Native"
replace race_selfid = "3" if race_selfid=="American Indian or Alaska Native"
replace race_selfid = "4" if race_selfid=="Native Hawaiian/Pacific Islander"
replace race_selfid = "4" if race_selfid=="Native Hawaiian or Pacific Islander"
replace race_selfid = "4" if race_selfid=="Native Hawaiian or Other Pacific Islander"
replace race_selfid = "5" if race_selfid=="Other"
replace race_selfid = "6" if race_selfid=="Two or More Races"
destring race_selfid, replace
label define race_selfid_lab 0 "White" 1 "Black or African American" 2 "Asian" 3 "American Indian or Alaska Native" 4 "Native Hawaiian or Other Pacific Islander" 5 "Other" 6 "Two or More Races"
label values race_selfid race_selfid_lab
label variable race_selfid "Race"

*Household Annual Income*
replace income = "0" if income=="Less than $10,000"
replace income = "1" if income=="$10,000 to $14,999"
replace income = "2" if income=="$15,000 to $24,999"
replace income = "3" if income=="$25,000 to $34,999"
replace income = "4" if income=="$35,000 to $49,999"
replace income = "5" if income=="$50,000 to $74,999"
replace income = "6" if income=="$75,000 to $99,999"
replace income = "7" if income=="$100,000 to $149,999"
replace income = "8" if income=="$150,000 to $199,999"
replace income = "9" if income=="$200,000 or more"
destring income, replace
label define income_lab 0 "Less than $10,000" 1 "$10,000 to $14,999" 2 "$15,000 to $24,999" 3 "$25,000 to $34,999" 4 "$35,000 to $49,999" 5 "$50,000 to $74,999" 6 "$75,000 to $99,999" 7 "$100,000 to $149,999" 8 "$150,000 to $199,999" 9 "$200,000 or more"
label values income income_lab
label variable income "Total Household Annual Income (before taxes)"
```

```

*Homeownership*
replace homeownership = "0" if homeownership=="Rent"
replace homeownership = "1" if homeownership=="Own"
destring homeownership, replace
label define homeownership_lab 0 "Rent" 1 "Own"
label values homeownership homeownership_lab
label variable homeownership "Homeownership"

*Rurality - only available for 2022 and 2023*
replace rurality = "0" if rurality=="Rural"
replace rurality = "1" if rurality=="Urban"
replace rurality = "1" if rurality=="Urban Cluster"
destring rurality, replace
label define rurality_lab 0 "Rural" 1 "Urban"
label values rurality rurality_lab
label variable rurality "Rurality Calculated using ZIP code, County, and State"

count

** Check for multicolinearity among independent variables**
correlate atleast3_prep_action_gen awareness_gen awareness_pan pan_exp pan_confidence pan_perception
race_selfid age homeownership education sex income census_region

*Table 1 - Weighted*
svyset _n, weight(weight) vce(linearized) singleunit(missing)

dtable i.atleast3_prep_action_gen i.awareness_gen i.awareness_pan i.pan_exp i.pan_confidence i.
pan_perception i.race_selfid i.age i.homeownership i.education i.sex i.income i.census_region, by(
atleast3_prep_action_pan) svy export(NHS2023_table1_weighted_bypandemicpreparednessstatus.docx,
replace)

*Descriptive Statistics - weighted*

*Mean number of disaster preparedness actions*
svy: mean num_prep_action_general
svy: tabulate num_prep_action_general

foreach var of varlist dis_prepactions_a dis_prepactions_b dis_prepactions_c dis_prepactions_d
dis_prepactions_e dis_prepactions_f dis_prepactions_g dis_prepactions_h dis_prepactions_i
dis_prepactions_j dis_prepactions_k dis_prepactions_l dis_prepactions_m dis_prepactions_n{
    svy: mean `var'
}

foreach var of varlist dis_prepactions_a dis_prepactions_b dis_prepactions_c dis_prepactions_d
dis_prepactions_e dis_prepactions_f dis_prepactions_g dis_prepactions_h dis_prepactions_i
dis_prepactions_j dis_prepactions_k dis_prepactions_l dis_prepactions_m dis_prepactions_n{
    svy: tabulate `var'
}

*Mean number of pandemic preparedness actions*
svy: mean num_prep_action_pandemic
svy: tabulate num_prep_action_pandemic

foreach var of varlist pan_prepactions_a pan_prepactions_b pan_prepactions_c pan_prepactions_d
pan_prepactions_e pan_prepactions_f pan_prepactions_g pan_prepactions_h pan_prepactions_i
pan_prepactions_j pan_prepactions_k pan_prepactions_l pan_prepactions_m pan_prepactions_n{
    svy: mean `var'
}

foreach var of varlist pan_prepactions_a pan_prepactions_b pan_prepactions_c pan_prepactions_d
pan_prepactions_e pan_prepactions_f pan_prepactions_g pan_prepactions_h pan_prepactions_i
pan_prepactions_j pan_prepactions_k pan_prepactions_l pan_prepactions_m pan_prepactions_n{
```

```
    svy: tabulate `var'
}

*Number of people with at least 3 disaster preparedness actions*
svy: mean atleast3_prep_action_gen
svy: tabulate atleast3_prep_action_gen

*Number of people with at least 3 pandemic preparedness actions*
svy: mean atleast3_prep_action_pan
svy: tabulate atleast3_prep_action_pan

*Mean pandemic experience*
svy: mean pan_exp
svy: tabulate pan_exp

*Mean awareness of disaster information*
svy: mean awareness_gen
svy: tabulate awareness_gen

foreach var of varlist dis_awareness_a dis_awareness_b dis_awareness_c dis_awareness_d dis_awareness_e dis_awareness_f dis_awareness_g dis_awareness_h dis_awareness_i dis_awareness_j dis_awareness_k dis_awareness_l {
    svy: mean `var'
}

foreach var of varlist dis_awareness_a dis_awareness_b dis_awareness_c dis_awareness_d dis_awareness_e dis_awareness_f dis_awareness_g dis_awareness_h dis_awareness_i dis_awareness_j dis_awareness_k dis_awareness_l {
    svy: tabulate `var'
}

*Mean awareness of pandemic information*
svy: mean awareness_pan
svy: tabulate awareness_pan

foreach var of varlist pan_awareness_a pan_awareness_b pan_awareness_c pan_awareness_d pan_awareness_e pan_awareness_f pan_awareness_g pan_awareness_h pan_awareness_i pan_awareness_j{
    svy: mean `var'
}

foreach var of varlist pan_awareness_a pan_awareness_b pan_awareness_c pan_awareness_d pan_awareness_e pan_awareness_f pan_awareness_g pan_awareness_h pan_awareness_i pan_awareness_j{
   svy: tabulate `var'
}

*Table 2 - Weighted Logistic Regression*

*At Least 3 Preapredness Actions within the Past Year with Indicator Variables Weighted - Pandemic Preparedness (Yes, No)*
quietly svy: logistic  atleast3_prep_action_pan atleast3_prep_action_gen, or
estimates store model1
quietly svy: logistic  atleast3_prep_action_pan awareness_gen, or
estimates store model2
quietly svy: logistic  atleast3_prep_action_pan awareness_pan, or
estimates store model3
quietly svy: logistic  atleast3_prep_action_pan pan_exp, or
estimates store model4
quietly svy: logistic  atleast3_prep_action_pan i.pan_confidence, or
estimates store model5
quietly svy: logistic  atleast3_prep_action_pan i.pan_perception, or
estimates store model6
quietly svy: logistic atleast3_prep_action_pan i.race_selfid, or
estimates store model7
```

```
quietly svy: logistic  atleast3_prep_action_pan i.age, or
estimates store model8
quietly svy: logistic atleast3_prep_action_pan homeownership, or
estimates store model9
quietly svy: logistic  atleast3_prep_action_pan i.education, or
estimates store model10
quietly svy: logistic atleast3_prep_action_pan i.sex, or
estimates store model11
quietly svy: logistic  atleast3_prep_action_pan i.income, or
estimates store model12
quietly svy: logistic  atleast3_prep_action_pan i.census_region, or
estimates store model13

**Multivariate Pandemic Preparedness Weighted - All Variables - No Interaction**
quietly svy: logistic atleast3_prep_action_pan atleast3_prep_action_gen awareness_gen awareness_pan pan_exp i.pan_confidence i.pan_perception i.race_selfid i.age homeownership i.education i.sex i.income i.census_region, or
est store A

etable, estimates(model1 model2 model3 model4 model5 model6 model7 model8 model9 model10 model11 model12 model13) showstars showstarsnote stars(0.001 "***" 0.01 "**" 0.05 "*") title("Table 2a: Univariate models for FEMA 2023") export(fema2023_nhs_unireg.docx, replace)

*Investigating Interaction Term - Disaster Preparedness and Census Region*
gen dis_prep_census_region=atleast3_prep_action_gen*census_region
label variable dis_prep_census_region "Interaction between Disaster Preparedness and Census Region"

** Multivariate Pandemic Preparedness Weighted - All Variables with Interaction**
quietly svy: logistic atleast3_prep_action_pan atleast3_prep_action_gen awareness_gen awareness_pan pan_exp i.pan_confidence i.pan_perception i.race_selfid i.age homeownership i.education i.sex i.income i.census_region dis_prep_census_region, or
est store B

** Only include covariates with at least one significant category from the univariate analysis**

* Homeownership was removed because none of the subcategories of these variables had a significant result for the univariate regression. *


**Multivariate Pandemic Preparedness Weighted Significant Covariates with Interaction*
quietly svy: logistic atleast3_prep_action_pan atleast3_prep_action_gen awareness_gen awareness_pan pan_exp i.pan_confidence i.pan_perception i.race_selfid i.age i.education i.sex i.income i.census_region dis_prep_census_region, or
est store C
estimates store model14
estat gof

* The interaction term was removed because it was not found to be significant across all study years *

**Multivariate Pandemic Preparedness Weighted Significant Covariates across all three years - removed homeownership, income, census region, and the interaction term**
svy: logistic atleast3_prep_action_pan atleast3_prep_action_gen awareness_gen awareness_pan pan_exp i.pan_confidence i.pan_perception i.race_selfid i.age i.education i.sex, or
estimates store model15
est store D
est stats *
estat gof

etable, estimates(model14 model15) showstars showstarsnote stars(0.001 "***" 0.01 "**" 0.05 "*") title("Table 2a: Multivariate models for FEMA 2023") export(fema2023_nhs_multireg.docx, replace)

save "C:\Location\fema_national_household_survey_2023_data_and_codebook_results.dta", replace
```

```
clear

*** Figure 1 ***

*Please note a excel sheet was created with the following data by year: mean preparedness actions (disaster and pandemic), the proportion of survey participants with at least 3 preparedness actions (disaster and pandemic), the proportion of survey participants with at least 3 preparedness actions (disaster and pandemic), the proportion of survey participants with awareness of pandemic or disaster related information, and the proportion of survey participants with previous pandemic. Disaster preparedness was indicated by "0" for the category variable. Pandemic preparedness was indicated by "1" for the category variable*

* Import an excel sheet with the data above*

mkdir graphs

replace category = "0" if category=="Disaster Preparedness"
replace category = "1" if category=="Pandemic Preparedness"

*Graph mean number of preparedness actions by year*
graph bar mean_num_prep_actions, over(category, gap(0)) over(year) ascategory asyvars bar(1, color(stc1)) bar(2, color(stc2)) title("Mean Count of Disaster and Pandemic Preparedness Actions" "Completed by Survey Participants within the Past Year", size(large) span margin(b=5)) ytitle( "Mean Count of Individual Preapredness Actions",  placement(center)) ylabel(, ang(h) labsize(medium)) legend(order(1 "Disaster Preparedness Actions" 2 "Pandemic Preparedness Actions")  size(medium) position(6) row(1) margin(medium))

graph save "graphs/mean_count_prep_actions2.gph", replace
graph export "graphs/mean_count_prep_action2.jpg", replace
graph export "graphs/mean_count_prep_action_maxres2.jpg", width(800) height(600) quality(100) replace

*Graph proportion of survey participants with at least 3 preparedness actions (prepared) by year*
graph bar prop_least_prep_actions, over(category, gap(0)) over(year) ascategory asyvars bar(1, color(stc1)) bar(2, color(stc2)) title("Proportion of NHS Participants Considered to be" "Prepared for a Disaster or Pandemic", size(large) span margin(b=0)) subtitle("{it:Preparedness is defined as completing at least 3 or more preparedness actions within the last year}", size(medsmall) span margin(b=5)) ytitle("Proportion of Survey Participants", placement(center)) ylabel(, format(%4.1f) ang(h) labsize(medium)) legend(order(1 "Prepared for a Disaster" 2 "Prepared for a Pandemic")  size(medium) position(6) row(1) margin(medium))

graph save "graphs/prop_least_prep_actions2.gph", replace
graph export "graphs/prop_least_prep_actions2.jpg", replace
graph export "graphs/prop_least_prep_actions_maxres2.jpg", width(800) height(600) quality(100) replace


*Graph proportion of survey participants with awareness of pandemic or disaster related information by year*
gen prop_awareness_percent = prop_awareness * 100
graph bar prop_awareness, over(category, gap(0)) over(year) ascategory asyvars bar(1, color(stc1)) bar(2, color(stc2)) title("Proportion of NHS Participants Indicating Awareness of" "Disaster or Pandemic-related Information", size(large) span margin(b=5)) ytitle("Proportion of Survey Participants", placement(center))  ylabel(, format(%4.1f) ang(h) labsize(medium)) legend(order(1 "Disaster Awareness" 2 "Pandemic Awareness")  size(medium) position(6) row(1) margin(medium))

graph save "graphs/prop_awareness2.gph", replace
graph export "graphs/prop_awareness2.jpg", replace
graph export "graphs/prop_awareness_maxres2.jpg", width(800) height(600) quality(100) replace

*Graph proportion of survey participants with previous pandemic experience by year*
graph bar prop_pan_exp,over(category, gap(0)) over(year) ascategory asyvars bar(1, color(stc2)) bar(2, color(stc2)) title("Proportion of NHS Participants with Previous Pandemic Experience", size(large) span margin(b=5)) ytitle("Proportion of Survey Participants", placement(center)) ylabel(, format(%4.1f) ang(h) labsize(medium)) legend(order(1 "Previous Pandemic Exerpience")  size(medium) position(6
```

```
) row(1) margin(medium))

graph save "graphs/prop_pan_exp2.gph", replace
graph export "graphs/prop_pan_exp2.jpg", replace
graph export "graphs/prop_pan_exp_maxres2.jpg", width(800) height(600) quality(100) replace

*Authors combined and formated these graphs using Publisher*

*** Figure 2 ***

clear

ssc install coefplot

*2021*
use "C:\Location\fema_national_household_survey_data_and_codebook_results_2021.dta"
quietly svy: logistic atleast3_prep_action_pan atleast3_prep_action_gen awareness_gen awareness_pan
genexpa i.c2_1g i.l1a i.race i.age i.educ i.gender, or
estimates store NHS_2021
clear

*2022*
use"C:\Location\run_FULL_04072025\fema_national_household_survey_2022_data_and_codebook_results.dta"
quietly svy: logistic atleast3_prep_action_pan atleast3_prep_action_gen awareness_gen awareness_pan
genexpa i.c2_1g i.l1a i.race i.age i.education i.sex, or
estimates store NHS_2022
clear

*2023*
use"C:\Location\fema_national_household_survey_2023_data_and_codebook_results.dta"
rename pan_exp genexpa
rename pan_confidence c2_1g
rename pan_perception l1a
rename race_selfid race
quietly svy: logistic atleast3_prep_action_pan atleast3_prep_action_gen awareness_gen awareness_pan
genexpa i.c2_1g i.l1a i.race i.age i.education i.sex, or
estimates store NHS_2023

*Combined Forest Plot*
coefplot (NHS_2021, drop(_cons) eform lcolor(blue) mcolor(blue) msymbol(O) keep(
atleast3_prep_action_gen awareness_gen awareness_pan genexpa 2.c2_1g 3.c2_1g 4.c2_1g 5.c2_1g 1.l1a
2.l1a)) (NHS_2022, drop(_cons) eform lcolor(red) mcolor(red) msymbol(O) keep(atleast3_prep_action_gen
 awareness_gen awareness_pan genexpa 2.c2_1g 3.c2_1g 4.c2_1g 5.c2_1g 1.l1a 2.l1a)) (NHS_2023, drop(
_cons) eform bcolor(green) mcolor(green) msymbol(O) keep(atleast3_prep_action_gen awareness_gen
awareness_pan genexpa 2.c2_1g 3.c2_1g 4.c2_1g 5.c2_1g 1.l1a 2.l1a)), title("Forest Plot by Year")
xscale(range(0 30)) xlabel(0(5)30) ylabel(, labs(small) angle(horizontal))

*Export graph*
graph save "graphs/stacked_forestplot_top6covariates.gph", replace
graph export "graphs/stacked_forestplot_top6covariates.png", replace

*Forest plot with no label*
coefplot (NHS_2021, drop(_cons) eform lcolor(blue) mcolor(blue) msymbol(O) keep(
atleast3_prep_action_gen awareness_gen awareness_pan genexpa 2.c2_1g 3.c2_1g 4.c2_1g 5.c2_1g 1.l1a
2.l1a)) (NHS_2022, drop(_cons) eform lcolor(red) mcolor(red) msymbol(O) keep(atleast3_prep_action_gen
 awareness_gen awareness_pan genexpa 2.c2_1g 3.c2_1g 4.c2_1g 5.c2_1g 1.l1a 2.l1a)) (NHS_2023, drop(
_cons) eform bcolor(green) mcolor(green) msymbol(O) keep(atleast3_prep_action_gen awareness_gen
awareness_pan genexpa 2.c2_1g 3.c2_1g 4.c2_1g 5.c2_1g 1.l1a 2.l1a)), title("Forest Plot by Year")
xscale(range(0 30)) xlabel(0(5)30) ylabel(, angle(horizontal)) legend(off)

*Export graph*
graph save "graphs/stacked_forestplot_top6covariates_nolabel.gph", replace
graph export "graphs/stacked_forestplot_top6covariates_nolabel.png", replace
```

```
*Format the layout of the graphs as you would like. Consider using a visualization software to edit and format the graphs.*

*** Determining the Number of Participants with identical selection of preparedness actions for disasters and pandemics ***

 clear all
**2021**
use "C:\Location\fema_national_household_survey_data_and_codebook_results_2021.dta"

* Step 1: Generate a new variable to track if all answers match
svyset _n, weight(weight) vce(linearized) singleunit(missing)
gen exact_same_answers = 1

* Step 2: Use a loop to compare each pair of disaster (prepa_*) and pandemic (prepb_*) variables
foreach var in prepa_a prepa_b prepa_c prepa_d prepa_e prepa_f prepa_g prepa_h prepa_i prepa_j prepa_k prepa_l prepa_m {
    * Map the corresponding pandemic variable by replacing "prepa" with "prepb"
    local pandemic_var = subinstr("`var'", "prepa", "prepb", .)

    * Check if the disaster and pandemic answers match; if not, set exact_same_answers to 0
    replace exact_same_answers = 0 if `var' != `pandemic_var'
}

* Step 3: Count the number of respondents with all matching answers
svy: tab exact_same_answers, percent
count if exact_same_answers == 1
clear all

**2022**

use "C:\Location\fema_national_household_survey_2022_data_and_codebook_results.dta"

* Step 1: Generate a new variable to track if all answers match
svyset _n, weight(weight) vce(linearized) singleunit(missing)
gen exact_same_answers = 1

* Step 2: Use a loop to compare each pair of disaster (prepa_*) and pandemic (prepb_*) variables
foreach var in prepa_a prepa_b prepa_c prepa_d prepa_e prepa_f prepa_g prepa_h prepa_i prepa_j prepa_k prepa_l prepa_m {
    * Map the corresponding pandemic variable by replacing "prepa" with "prepb"
    local pandemic_var = subinstr("`var'", "prepa", "prepb", .)

    * Check if the disaster and pandemic answers match; if not, set exact_same_answers to 0
    replace exact_same_answers = 0 if `var' != `pandemic_var'
}

* Step 3: Count the number of respondents with all matching answers
svy: tab exact_same_answers, percent
count if exact_same_answers == 1
clear all

**2023*
use "C:\Location\fema_national_household_survey_2023_data_and_codebook_results.dta"

* Step 1: Generate a new variable to track if all answers match
svyset _n, weight(weight) vce(linearized) singleunit(missing)
gen exact_same_answers = 1

* Step 2: Use a loop to compare each pair of disaster (prepa_*) and pandemic (prepb_*) variables
foreach var in dis_prepactions_a dis_prepactions_b dis_prepactions_c dis_prepactions_d
```

```
dis_prepactions_e dis_prepactions_f dis_prepactions_g dis_prepactions_h dis_prepactions_i
dis_prepactions_j dis_prepactions_k dis_prepactions_l dis_prepactions_m dis_prepactions_n{
    * Map the corresponding pandemic variable by replacing "dis_prepactions" with "pan_prepactions"
    local pandemic_var = subinstr("`var'", "dis_prepactions", "pan_prepactions", .)

    * Check if the disaster and pandemic answers match; if not, set exact_same_answers to 0
    replace exact_same_answers = 0 if `var' != `pandemic_var'
}

* Step 3: Count the number of respondents with all matching answers
svy: tab exact_same_answers, percent
count if exact_same_answers == 1
```
