# Supplementary material for: The Positive Influence of Individual-Level Disaster Preparedness on the Odds of Individual-Level Pandemic Preparedness—Insights from FEMA’s 2021–2023 National Household Survey
Source: Int J Environ Res Public Health. 2025 Apr 29;22(5):702. doi: 10.3390/ijerph22050702 (PMC12111411; doi:10.3390/ijerph22050702)
Supplement: Supplementary file 1 [file ijerph-22-00702-s001.zip › ijerph-3522834_SuplementalMaterials_TableS1&S2_04262025.pdf]

Table S1. Weighted Sample Characteristics of 2021-2023 FEMA National Household Survey Participants Stratified by Individual Pandemic Preparedness Status[a]

Survey Year	2021			2022			2023		
	Unprepared	Prepared	Total	Unprepared	Prepared	Total	Unprepared	Prepared	Total
N	2907 (46.0%)	3414 (54.0%)	6321	3498 (56.7%)	2673 (43.3%)	6171	3757 (58.7%)	2647 (41.3%)	6404
At Least 3 Disaster Preparedness Actions in the Past Year [b]									
Unprepared	2082 (71.6%)	319 (9.4%)	2401 (38.0%)	2215 (63.3%)	360 (13.5%)	2576 (41.7%)	2157 (57.4%)	345 (13.0%)	2502 (39.1%)
Prepared	825 (28.4%)	3095 (90.6%)	3920 (62.0%)	1283 (36.7%)	2313 (86.5%)	3596 (58.3%)	1599 (42.6%)	2303 (87.0%)	3902 (60.9%)
Awareness of Disaster Preparedness Information									
No	377 (13.0%)	57 (1.7%)	434 (6.9%)	503 (14.4%)	72 (2.7%)	575 (9.3%)	507 (13.5%)	42 (1.6%)	549 (8.6%)
Yes	2530 (87.0%)	3357 (98.3%)	5887 (93.1%)	2995 (85.6%)	2601 (97.3%)	5597 (90.7%)	3250 (86.5%)	2605 (98.4%)	5855 (91.4%)
Awareness of Pandemic Preparedness Information									
No	161 (5.5%)	23 (0.7%)	183 (2.9%)	235 (6.7%)	27 (1.0%)	262 (4.2%)	331 (8.8%)	31 (1.2%)	362 (5.6%)
Yes	2746 (94.5%)	3392 (99.3%)	6138 (97.1%)	3263 (93.3%)	2647 (99.0%)	5909 (95.8%)	3426 (91.2%)	2616 (98.8%)	6042 (94.4%)
Previous Pandemic Experience									
No	1120 (38.8%)	912 (26.8%)	2033 (32.3%)	1826 (52.2%)	1009 (37.7%)	2835 (45.9%)	912 (24.3%)	493 (18.6%)	1406 (21.9%)
Yes	1768 (61.2%)	2496 (73.2%)	4264 (67.7%)	1672 (47.8%)	1665 (62.3%)	3337 (54.1%)	2844 (75.7%)	2154 (81.4%)	4998 (78.1%)
Pandemic Confidence/Self-efficacy									
Not at all confident	137 (4.7%)	50 (1.5%)	186 (2.9%)	193 (5.5%)	26 (1.0%)	218 (3.5%)	152 (4.0%)	20 (0.8%)	172 (2.7%)
Slightly confident	465 (16.0%)	304 (8.9%)	769 (12.2%)	320 (9.1%)	94 (3.5%)	414 (6.7%)	335 (8.9%)	76 (2.9%)	411 (6.4%)
Somewhat confident	905 (31.1%)	721 (21.1%)	1626 (25.7%)	1025 (29.3%)	484 (18.1%)	1509 (24.4%)	981 (26.1%)	387 (14.6%)	1368 (21.4%)
Moderately confident	883 (30.4%)	1209 (35.4%)	2092 (33.1%)	1140 (32.6%)	997 (37.3%)	2136 (34.6%)	1271 (33.8%)	979 (37.0%)	2250 (35.1%)
Extremely confident	517 (17.8%)	1132 (33.1%)	1649 (26.1%)	820 (23.5%)	1073 (40.1%)	1894 (30.7%)	1019 (27.1%)	1184 (44.7%)	2203 (34.4%)
Pandemic Risk Perception									
Unlikely	439 (15.1%)	335 (9.8%)	773 (12.2%)	633 (18.1%)	289 (10.8%)	922 (14.9%)	765 (20.4%)	330 (12.4%)	1094 (17.1%)
Likely	1285 (44.2%)	1223 (35.8%)	2508 (39.7%)	1522 (43.5%)	1006 (37.6%)	2528 (41.0%)	1755 (46.7%)	1069 (40.4%)	2824 (44.1%)
Very likely	1184 (40.7%)	1856 (54.4%)	3040 (48.1%)	1344 (38.4%)	1379 (51.6%)	2722 (44.1%)	1237 (32.9%)	1249 (47.2%)	2485 (38.8%)
Race									
White	2154 (74.1%)	2557 (74.9%)	4711 (74.5%)	2570 (73.5%)	1882 (70.4%)	4452 (72.1%)	2643 (70.4%)	1758 (66.4%)	4401 (68.7%)
Black or African American	371 (12.8%)	404 (11.8%)	776 (12.3%)	413 (11.8%)	378 (14.2%)	791 (12.8%)	418 (11.1%)	345 (13.0%)	763 (11.9%)
Asian	187 (6.4%)	156 (4.6%)	344 (5.4%)	224 (6.4%)	137 (5.1%)	361 (5.9%)	227 (6.0%)	143 (5.4%)	369 (5.8%)
American Indian or Alaska Native	30 (1.0%)	20 (0.6%)	49 (0.8%)	29 (0.8%)	32 (1.2%)	61 (1.0%)	32 (0.9%)	23 (0.9%)	55 (0.9%)
Native Hawaiian or Other Pacific Islander	7 (0.2%)	6 (0.2%)	13 (0.2%)	7 (0.2%)	7 (0.3%)	13 (0.2%)	6 (0.2%)	8 (0.3%)	13 (0.2%)
Other	109 (3.7%)	174 (5.1%)	283 (4.5%)	172 (4.9%)	175 (6.6%)	347 (5.6%)	187 (5.0%)	161 (6.1%)	349 (5.4%)
Two or More Races	49 (1.7%)	97 (2.8%)	146 (2.3%)	83 (2.4%)	62 (2.3%)	146 (2.4%)	244 (6.5%)	210 (7.9%)	454 (7.1%)
Age [c]									
18-29	580 (20.0%)	710 (20.8%)	1290 (20.4%)	731 (20.9%)	576 (21.5%)	1307 (21.2%)	730 (19.4%)	569 (21.5%)	1299 (20.3%)
30-39	395 (13.6%)	686 (20.1%)	1081 (17.1%)	506 (14.5%)	564 (21.1%)	1070 (17.3%)	534 (14.2%)	652 (24.6%)	1186 (18.5%)
40-49	426 (14.7%)	610 (17.9%)	1036 (16.4%)	555 (15.9%)	452 (16.9%)	1008 (16.3%)	544 (14.5%)	503 (19.0%)	1047 (16.3%)
50-59	549 (18.9%)	546 (16.0%)	1095 (17.3%)	659 (18.8%)	381 (14.3%)	1040 (16.8%)	697 (18.5%)	375 (14.2%)	1071 (16.7%)
60-69	467 (16.1%)	483 (14.2%)	951 (15.0%)	561 (16.0%)	347 (13.0%)	907 (14.7%)	668 (17.8%)	298 (11.3%)	966 (15.1%)
70-79	306 (10.5%)	251 (7.4%)	557 (8.8%)	317 (9.1%)	209 (7.8%)	526 (8.5%)	398 (10.6%)	152 (5.7%)	550 (8.6%)
Over 80	183 (6.3%)	128 (3.8%)	312 (4.9%)	170 (4.9%)	144 (5.4%)	314 (5.1%)	187 (5.0%)	98 (3.7%)	285 (4.4%)
Homeownership									
Rent	1017 (35.0%)	1076 (31.5%)	2093 (33.1%)	1215 (34.7%)	954 (35.7%)	2169 (35.2%)	1208 (32.1%)	806 (30.5%)	2014 (31.5%)

Own	1890 (65.0%)	2338 (68.5%)	4228 (66.9%)	2283 (65.3%)	1719 (64.3%)	4002 (64.8%)	2549 (67.9%)	1841 (69.5%)	4390 (68.5%)
Education									
Less than high school diploma	349 (12.0%)	308 (9.0%)	657 (10.4%)	427 (12.2%)	233 (8.7%)	660 (10.7%)	363 (9.7%)	211 (8.0%)	574 (9.0%)
High school degree or diploma	802 (27.6%)	864 (25.3%)	1666 (26.4%)	961 (27.5%)	671 (25.1%)	1632 (26.4%)	1089 (29.0%)	537 (20.3%)	1626 (25.4%)
Some college no degree	692 (23.8%)	773 (22.7%)	1465 (23.2%)	817 (23.4%)	623 (23.3%)	1440 (23.3%)	803 (21.4%)	505 (19.1%)	1308 (20.4%)
Associate's degree	239 (8.2%)	282 (8.3%)	521 (8.2%)	294 (8.4%)	218 (8.1%)	512 (8.3%)	314 (8.4%)	246 (9.3%)	560 (8.8%)
Bachelor's degree	564 (19.4%)	696 (20.4%)	1261 (19.9%)	654 (18.7%)	554 (20.7%)	1208 (19.6%)	816 (21.7%)	602 (22.8%)	1418 (22.2%)
Post graduate work/degree or professional degree	261 (9.0%)	490 (14.4%)	751 (11.9%)	344 (9.8%)	375 (14.0%)	719 (11.7%)	372 (9.9%)	545 (20.6%)	917 (14.3%)
Gender									
Male	1373 (47.2%)	1757 (51.5%)	3130 (49.5%)	1620 (46.3%)	1417 (53.0%)	3037 (49.2%)	1851 (49.3%)	1504 (56.8%)	3355 (52.4%)
Female	1512 (52.0%)	1641 (48.1%)	3152 (49.9%)	1859 (53.1%)	1242 (46.4%)	3100 (50.2%)	1889 (50.3%)	1127 (42.6%)	3016 (47.1%)
Third Gender/Other	22 (0.8%)	17 (0.5%)	39 (0.6%)	19 (0.5%)	15 (0.6%)	34 (0.5%)	16 (0.4%)	16 (0.6%)	32 (0.5%)
Total Household Annual Income (before taxes) [d]									
Less than $10,000	193 (6.6%)	164 (4.8%)	357 (5.6%)	201 (5.7%)	156 (5.8%)	357 (5.8%)	193 (5.1%)	105 (4.0%)	298 (4.6%)
$10,000 to $19,999 *2023:* $10,000 to $14,999	260 (8.9%)	262 (7.7%)	522 (8.3%)	345 (9.9%)	196 (7.3%)	541 (8.8%)	148 (3.9%)	87 (3.3%)	235 (3.7%)
$20,000 to $29,999 *2023:* $15,000 to $24,999	273 (9.4%)	291 (8.5%)	564 (8.9%)	308 (8.8%)	219 (8.2%)	527 (8.5%)	285 (7.6%)	183 (6.9%)	468 (7.3%)
$30,000 to $39,999 *2023:* $25,000 to $34,999	278 (9.6%)	250 (7.3%)	528 (8.4%)	300 (8.6%)	217 (8.1%)	517 (8.4%)	333 (8.9%)	188 (7.1%)	521 (8.1%)
$40,000 to $49,999 *2023:* $35,000 to $49,999	238 (8.2%)	278 (8.1%)	516 (8.2%)	274 (7.8%)	210 (7.8%)	483 (7.8%)	487 (13.0%)	261 (9.9%)	748 (11.7%)
$50,000 to $59,999 *2023:* $50,000 to $74,999	237 (8.2%)	221 (6.5%)	458 (7.2%)	246 (7.0%)	201 (7.5%)	447 (7.2%)	674 (17.9%)	412 (15.6%)	1085 (16.9%)
$60,000 to $99,999 *2023:* $75,000 to $99,999	667 (22.9%)	768 (22.5%)	1435 (22.7%)	787 (22.5%)	617 (23.1%)	1404 (22.8%)	475 (12.6%)	332 (12.5%)	806 (12.6%)
$100,000 to $149,999	388 (13.4%)	605 (17.7%)	994 (15.7%)	531 (15.2%)	442 (16.5%)	973 (15.8%)	579 (15.4%)	504 (19.0%)	1083 (16.9%)
$150,000 or more *2023:* $150,000 to $199,999	371 (12.8%)	575 (16.9%)	947 (15.0%)	505 (14.4%)	416 (15.6%)	922 (14.9%)	291 (7.8%)	234 (8.9%)	526 (8.2%)
2023: $200,000 or more							293 (7.8%)	341 (12.9%)	634 (9.9%)
Census Region									
West	1097 (37.7%)	1254 (36.7%)	2351 (37.2%)	1072 (30.6%)	853 (31.9%)	1925 (31.2%)	856 (22.8%)	652 (24.6%)	1508 (23.5%)
Midwest	614 (21.1%)	625 (18.3%)	1239 (19.6%)	703 (20.1%)	474 (17.7%)	1177 (19.1%)	849 (22.6%)	466 (17.6%)	1315 (20.5%)
Northeast	339 (11.7%)	529 (15.5%)	867 (13.7%)	613 (17.5%)	563 (21.1%)	1176 (19.1%)	667 (17.8%)	484 (18.3%)	1151 (18.0%)
South	833 (28.6%)	964 (28.2%)	1797 (28.4%)	1093 (31.3%)	769 (28.8%)	1862 (30.2%)	1352 (36.0%)	993 (37.5%)	2345 (36.6%)
Territories	25 (0.8%)	42 (1.2%)	67 (1.1%)	17 (0.5%)	14 (0.5%)	31 (0.5%)	33 (0.9%)	52 (2.0%)	85 (1.3%)

The table above was weighted using FEMA's geography-based weighting scheme. For all categorical variables included in the table above, the summary statistics are displayed in the n (%) format. Throughout the table within group percentages might add up to slightly more than 100 due to rounding.[a] *Level of pandemic preparedness was categorized into unprepared (an individual achieved less than 3 pandemic preparedness activities within the past year) and prepared (an individual has achieved 3 or more pandemic preparedness activities within the past year).*[b] *Level of disaster preparedness was categorized into unprepared (an individual has achieved less than 3 disaster preparedness activities within the past year) and prepared (an individual has achieved 3 or more disaster preparedness activities within the past year).*[c] *Age was only included as a categorical variable within the publicly available FEMA NHS dataset.*[d] *Alternative Total Household Annual Income categories were utilized during the 2023 NHS compared to the previous NHS conducted in 2021 and 2022.*

Table S2. Predictors of Individual-level Pandemic Preparedness via Weighted Univariate and Multivariate Logistic Regressions[a]

**Survey year**		**2021**			**2022**			**2023**	
	**Model 1**	**Model 2**	**Model 3**	**Model 1**	**Model 2**	**Model 3**	**Model 1**	**Model 2**	**Model 3**
Regression Type [b]	OR(SE)	OR(SE)	OR(SE)	OR(SE)	OR(SE)	OR(SE)	OR(SE)	OR(SE)	OR(SE)
Prepared for a	24.46***	17.97***	21.35***	11.08***	11.69***	9.26***	9.01***	9.16***	6.75***
Disaster [c] *(Ref: No)*	(2.85)	(4.55)	(2.59)	(0.96)	(2.42)	(0.87)	(0.74)	(1.83)	(0.59)
Awareness of Disaster Preparedness Information *(Ref:*	8.78***	3.06***	3.16***	6.06***	1.61*	1.62**	9.58***	3.02***	3.04***
No)	(2.19)	(0.97)	(1.00)	(1.00)	(0.30)	(0.30)	(2.13)	(0.74)	(0.73)
Awareness of Pandemic Preparedness Information *(Ref:*	8.80***	2.67	2.56	7.17***	2.79***	2.80***	8.10***	3.79***	3.78***
No)	(3.82)	(1.78)	(1.69)	(1.62)	(0.73)	(0.74)	(1.84)	(0.98)	(0.98)
Previous Pandemic	1.73***	1.28*	1.29*	1.80***	1.55***	1.56***	1.40***	1.01	1.01
Experience *(Ref: No)*	(0.15)	(0.15)	(0.15)	(0.12)	(0.13)	(0.13)	(0.11)	(0.10)	(0.10)
Pandemic Confidence/Self-efficacy *(Ref: Not at all confident)*									
	1.80	1.22	1.20	2.20**	1.71	1.73	1.69	1.37	1.36
Slightly confident	(0.55)	(0.44)	(0.44)	(0.61)	(0.56)	(0.57)	(0.55)	(0.54)	(0.53)
Somewhat	2.20**	1.38	1.36	3.53***	2.42**	2.44**	2.93***	1.84	1.83
confident	(0.64)	(0.48)	(0.48)	(0.89)	(0.70)	(0.71)	(0.89)	(0.68)	(0.68)
Moderately	3.78***	1.74	1.67	6.54***	3.56***	3.61***	5.72***	2.68**	2.65**
confident	(1.09)	(0.59)	(0.58)	(1.61)	(1.02)	(1.03)	(1.71)	(0.98)	(0.97)
Extremely	6.04***	2.87**	2.79**	9.79***	4.49***	4.52***	8.63***	3.41***	3.36***
confident	(1.76)	(0.99)	(0.99)	(2.41)	(1.29)	(1.30)	(2.58)	(1.25)	(1.23)
Pandemic Risk Perception *(Ref: Unlikely)*									
	1.25	0.92	0.91	1.45***	1.37*	1.36*	1.41***	1.18	1.16
Likely	(0.17)	(0.17)	(0.17)	(0.16)	(0.18)	(0.18)	(0.13)	(0.14)	(0.14)
	2.06***	1.06	1.08	2.25***	1.65***	1.65***	2.34***	1.53***	1.55***
Very likely	(0.28)	(0.20)	(0.20)	(0.24)	(0.21)	(0.21)	(0.22)	(0.19)	(0.19)
Race *(Ref: White)*									
Black or African	0.92	1.00	0.99	1.25*	1.31*	1.30*	1.24*	1.23	1.23
American	(0.12)	(0.18)	(0.18)	(0.13)	(0.17)	(0.17)	(0.12)	(0.14)	(0.14)
	0.70	0.76	0.76	0.84	0.91	0.94	0.95	0.98	1.04
Asian	(0.13)	(0.19)	(0.19)	(0.15)	(0.18)	(0.19)	(0.14)	(0.17)	(0.18)
American Indian or	0.56	0.46	0.46	1.49	1.49	1.50	1.08	1.32	1.37
Alaska Native	(0.25)	(0.22)	(0.22)	(0.49)	(0.58)	(0.58)	(0.17)	(0.23)	(0.23)
Native Hawaiian or Other Pacific	0.74	0.55	0.55	1.37	1.19	1.23	1.95**	1.90	2.01
Islander	(0.50)	(0.43)	(0.43)	(0.65)	(0.88)	(0.88)	(0.49)	(0.72)	(0.74)
	1.35	1.34	1.21	1.39	1.29	1.33	1.30	1.25	1.33
Other	(0.28)	(0.39)	(0.34)	(0.24)	(0.25)	(0.26)	(0.19)	(0.23)	(0.24)
	1.66*	2.12*	1.94	1.02	1.02	1.02	1.29	1.29	1.31
Two or More Races	(0.43)	(0.70)	(0.66)	(0.21)	(0.26)	(0.26)	(0.17)	(0.21)	(0.21)
Age *(Ref: 18-29)*									
	1.42**	1.36	1.35	1.42**	1.35*	1.34*	1.57***	1.26*	1.26*
30-39	(0.19)	(0.25)	(0.25)	(0.15)	(0.17)	(0.17)	(0.15)	(0.14)	(0.14)
40-49	1.17	0.91	0.91	1.03	1.00	1.00	1.19	1.03	1.03

	(0.16)	(0.19)	(0.18)	(0.12)	(0.14)	(0.14)	(0.12)	(0.13)	(0.13)
	0.81	0.61**	0.64*	0.73**	0.82	0.82	0.69***	0.73*	0.73*
50-59	(0.11)	(0.11)	(0.12)	(0.09)	(0.11)	(0.11)	(0.08)	(0.09)	(0.09)
	0.85	0.62*	0.63*	0.78*	0.91	0.92	0.57***	0.64***	0.64***
60-69	(0.12)	(0.12)	(0.12)	(0.09)	(0.13)	(0.13)	(0.06)	(0.08)	(0.08)
	0.67*	0.53**	0.53**	0.83	0.92	0.93	0.49***	0.57***	0.57***
70-79	(0.11)	(0.11)	(0.11)	(0.11)	(0.14)	(0.15)	(0.06)	(0.08)	(0.08)
	0.57*	0.53	0.52*	1.08	1.38	1.40	0.67*	0.83	0.83
Over 80	(0.14)	(0.18)	(0.17)	(0.24)	(0.37)	(0.38)	(0.11)	(0.18)	(0.18)
Homeownership	1.17			0.96			1.08		
(Ref: Rent)	(0.10)			(0.07)			(0.07)		
Education Status *(Ref: Less than high school diploma)*									
High school degree	1.22	1.04	1.06	1.28	1.26	1.26	0.85	0.71	0.71
or diploma	(0.23)	(0.26)	(0.26)	(0.22)	(0.25)	(0.25)	(0.14)	(0.15)	(0.15)
Some college, no	1.27	0.99	1.01	1.40*	1.22	1.22	1.08	1.01	0.99
degree	(0.24)	(0.24)	(0.24)	(0.24)	(0.24)	(0.24)	(0.17)	(0.22)	(0.21)
	1.34	1.02	1.06	1.36	1.19	1.20	1.35	1.08	1.07
Associate's degree	(0.28)	(0.28)	(0.28)	(0.25)	(0.26)	(0.26)	(0.23)	(0.25)	(0.23)
	1.40	0.98	1.05	1.55*	1.11	1.13	1.27	1.01	1.01
Bachelor's degree	(0.26)	(0.25)	(0.26)	(0.27)	(0.22)	(0.23)	(0.20)	(0.23)	(0.21)
Post graduate									
work/degree or	2.13***	0.91	1.02	2.00***	1.30	1.32	2.52***	1.45	1.50
professional degree	(0.42)	(0.25)	(0.26)	(0.36)	(0.27)	(0.27)	(0.43)	(0.34)	(0.32)
Gender *(Ref: Male)*									
	0.85*	0.84	0.83	0.76***	0.79**	0.78**	0.73***	0.79**	0.79**
Female	(0.07)	(0.10)	(0.09)	(0.05)	(0.06)	(0.06)	(0.05)	(0.06)	(0.06)
Third	0.58	1.61	1.56	0.88	0.89	0.90	1.18	1.32	1.43
Gender/Other	(0.30)	(0.98)	(0.95)	(0.42)	(0.40)	(0.41)	(0.49)	(0.80)	(0.84)
Total Household Annual Income (before taxes) *(Ref: Less than $10,000)*									
$10,000 to $19,999									
2023: $10,000 to	1.19	1.34		0.73			1.09	1.33	
$14,999	(0.27)	(0.38)		(0.14)			(0.21)	(0.30)	
$20,000 to $29,999									
2023: $15,000 to	1.26	1.08		0.92			1.18	1.38	
$24,999	(0.28)	(0.32)		(0.16)			(0.20)	(0.30)	
$30,000 to $39,999									
2023: $25,000 to	1.06	0.95		0.94			1.03	1.14	
$34,999	(0.24)	(0.28)		(0.16)			(0.17)	(0.24)	
$40,000 to $49,999									
2023: $35,000 to	1.38	1.03		0.99			0.99	1.00	
$49,999	(0.31)	(0.31)		(0.17)			(0.15)	(0.21)	
$50,000 to $59,999									
2023: $50,000 to	1.10	0.89		1.05			1.12	1.03	
$74,999	(0.25)	(0.27)		(0.18)			(0.16)	(0.21)	
$60,000 to $99,999	1.36	1.18		1.01			1.29	1.03	
	(0.27)	(0.31)		(0.16)			(0.19)	(0.22)	

2023: $75,000 to									
$99,999									
	1.84**	1.35		1.07			1.60**	1.13	
$100,000 to $149,999	(0.39)	(0.39)		(0.17)			(0.28)	(0.24)	
$150,000 or more									
2023: $150,000 to	1.83**	1.26		1.06			1.48*	0.92	
$199,999	(0.39)	(0.39)		(0.18)			(0.25)	(0.21)	
2023: $200,000 or							2.14***	1.33	
more							(0.39)	(0.33)	
Census Region *(Ref: West)*									
	0.89	1.05		0.85	1.04		0.72***	0.89	
Midwest	(0.10)	(0.19)		(0.08)	(0.14)		(0.07)	(0.11)	
	1.37*	1.40		1.16	1.22		0.95	1.07	
Northeast	(0.18)	(0.30)		(0.12)	(0.20)		(0.09)	(0.16)	
	1.01	0.76		0.88	1.02		0.96	1.10	
South	(0.10)	(0.18)		(0.08)	(0.20)		(0.07)	(0.19)	
	1.50	0.38		1.01	0.96		2.04***	1.76	
Territories	(0.59)	(0.20)		(0.48)	(0.51)		(0.38)	(0.51)	
Interaction between Disaster Preparedness and Census Region *(Ref: No interaction)*		1.10			0.91			0.90	
		(0.10)			(0.07)			(0.06)	
		0.01***	0.01***		0.01***	0.01***		0.01***	0.01***
Intercept		(0.01)	(0.01)		(0.00)	(0.00)		(0.00)	(0.00)

*** p<0.001, ** p<.01, * p<.05

The table above was weighted using FEMA's geography-based weighting scheme. [a] The level of pandemic preparedness was categorized into unprepared (an individual has achieved less than 3 pandemic preparedness activities within the past year) and prepared (an individual has achieved 3 or more pandemic preparedness activities within the past year). [b] For each survey year, regression type is defined as univariate logistic regression (model 1), multivariate logistic regression of significant covariates unique to each survey year (model 2), and multivariate logistic regression of significant covariates across all three survey years (model 3). [c] Level of disaster preparedness was categorized into unprepared (an individual has achieved less than 3 disaster preparedness activities within the past year) and prepared (an individual has achieved 3 or more disaster preparedness activities within the past year).
